# Integrated Metabolomics and Network Pharmacology Study on the Mechanism of Kangfuxiaoyan Suppository for Treating Chronic Pelvic Inflammatory Disease

**DOI:** 10.3389/fphar.2022.812587

**Published:** 2022-02-04

**Authors:** Zhengyi Zhang, Ziye Xie, Shujing Lv, Yulian Shi, Chuanjia Zhai, Xuejiao Li, Bin Qiao, Xiaoyan Gao

**Affiliations:** ^1^ School of Chinese Materia Medica, Beijing University of Chinese Medicine, Beijing, China; ^2^ Sunflower Pharmaceutical Group Co., Ltd, Beijing, China

**Keywords:** kangfuxiaoyan suppository, chronic pelvic inflammatory disease, UPLC-Q-TOF/MS, network pharmacology, metabolomics

## Abstract

Kangfuxiaoyan suppository (KFXYS) is a commonly used traditional Chinese medicine (TCM) preparation for the treatment of chronic pelvic inflammatory disease (CPID) clinically, and its safety and effectiveness have been well verified. However, the potential mechanism remains unclear. The integrated strategy of metabolomics and network pharmacology was employed in the study to reveal the potential mechanism of KFXYS in the treatment of CPID. Our research consists of five steps. First, the effect of KFXYS in reversing uterine inflammation indexes was verified. Second, based on the comprehensive characterization of 123 chemical ingredients of KFXYS, the ingredients of KFXYS absorbed into blood were identified by UPLC-Q-TOF/MS, then ADME research was carried out on the main ingredients. Third, the differential metabolites with significant correlation to inflammatory indexes were discovered by metabolomics and correlation analysis. Fourth, the potential targets and pathways of KFXYS in treating CPID were predicted by network pharmacology based on the ingredients which had good ADME behavior. Fifth, the proteins in common pathways of metabolomics and network pharmacology were used to screen the key targets from the potential targets of network pharmacology, and the potential mechanism of KFXYS in treating CPID was clarified. As a result, KFXYS significantly reversed the uterine inflammation indexes, including IL-1 and IL-6. The ingredients absorbed into blood including matrine, sophocarpine, aloin, esculetin-*O*-glucuronide, 7,4′-dihydroxyisoflavone-*O*-glucuronide, and 4′-methoxyisoflavone-7-*O*-glucuronide had good ADME behavior *in vivo*. Among the differential metabolites, Leukotriene A4, 5-Hydroxyindoleacetic acid, Ornithine, Arginine, and PC (20:1 (11Z)/20:4 (8Z,11Z,14Z,17Z)) were significant correlation to inflammation indexes. The integration analysis of metabolomics and network pharmacology shows that KFXYS may regulate the key targets including ARG1, NOS2, NOS3, etc. We speculate that ingredients of KFXYS, such as matrine, sophocarpine, aloin etc. act on the key proteins including ARG1, NOS2, and NOS3, to exert anti-inflammatory effect.

## Introduction

Chronic pelvic inflammatory disease (CPID) is a chronic inflammation of female pelvic reproductive organs, surrounding connective tissue and pelvic peritoneum (Cao, 2008), which can lead to infertility, ectopic pregnancy, or chronic pelvic pain (Soper, 2010). Antibiotics such as levofloxacin or metronidazole are mostly used in treating CPID (Duarte et al., 2015), but the therapeutic effect is not ideal due to drug resistance and side effects. Therefore, it is necessary to develop new alternative drugs to treat CPID.

Kangfuxiaoyan suppository (KFXYS) is a Chinese patent medicine commonly used for the treatment of CPID (Standardized project group of "Clinical Application Guidelines of the Chinese Medicine for Treatment of Common Disease, 2020). The formula of KFXYS is composed of Sophorae Flavescentis Radix (SFR, derived from *Sophora flavescens* Aiton.), Andrographis Herba [AH, derived from *Andrographis paniculata* (Burm.f.) Nees], Arnebiae Radix [AR, derived from *Arnebia euchroma* (Royle ex Benth.) I.M.Johnst.], Herba Patriniae (HP, *Patrinia villosa* (Thunb.) Dufr. or *Patrinia scabiosifolia* Link), Taraxaci Herba (TH, derived from *Taraxacum mongolicum* Hand.-Mazz. or *Taraxacum sinicum* Kitag.), Violsse Herba (VH, derived from *Viola philippica* Cav.), Aloe [Aloe, derived from *Aloe vera* (L.) Burm. f. or *Aloe ferox* Mill.], and Suis Fellis Pulvis (SFP, derived from *Sus scrofa domestica* Brisson.) (Chinese Pharmacopoeia Commission, [CPC], 2020). Our previous study has shown that KFXYS could alleviate the degree of uterine swelling in CPID, inhibit the secretion of inflammatory factors, and increase the immune organ index, the efficacy of KFXYS was verified in rat with CPID (Xie et al., 2018), but the underlying mechanism is still unknown.

In order to reveal the mechanism of the synergistic effect of multi-components of TCM, integrated metabolomics and network pharmacology strategy has been successfully used (Li et al., 2021). In recent years, metabolomics has been successfully applied to the study of the mechanism of TCM in treating diseases (Geiger et al., 2016). However, due to the lack of specificity of non-targeted metabolomics research, the discovery of differential metabolites correlation with pharmacodynamic indicators contribute to the following mechanism research. Network pharmacology can explore the changes of targets and pathways *in vivo* caused by diseases, and the disturbance of one or more nodes in the biological network after TCM preparation acting on the body (Hopkins, 2008), so as to effectively establish a “compound-protein/gene-disease” network, reveal the regulation principles of small molecules in a high-throughput manner (Zhang et al., 2019). However, we believe that the results of network pharmacology based on the components absorbed into blood which had good ADME behavior is more accurate and reliable.

**FIGURE 1:**
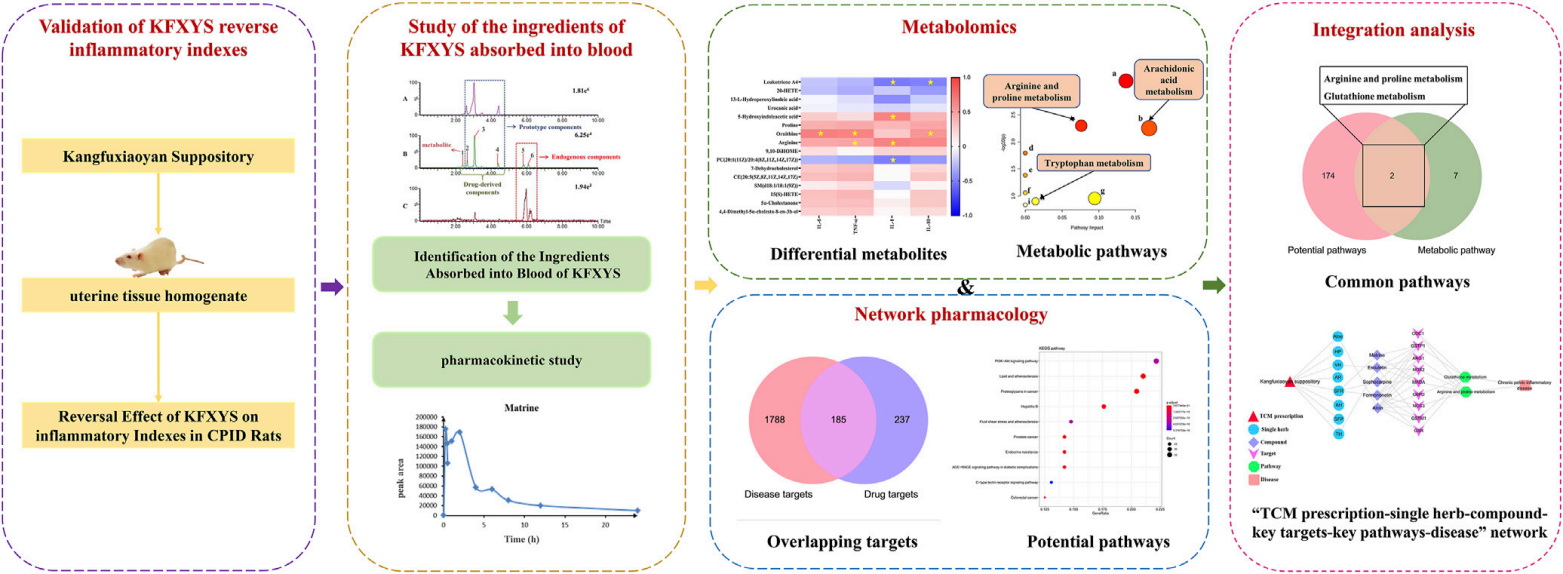
Schematic diagram of the action mechanism of KFXYS in treating CPID.

In this study, the reversal effect of KFXYS on inflammatory indexes was verified in CPID model rats. Based on clarifying the chemical ingredients of KFXYS, the ingredient of KFXYS absorbed into blood were further characterized by UPLC-Q-TOF/MS technology, then ADME research was carried out. Among the differential metabolites discovered by metabolomics, the metabolites with significant correlation to inflammatory indexes were discovered by correlation analysis. Then based on the ingredients absorbed into blood which had good ADME behavior, network pharmacology was used to predict the potential targets and pathways of KFXYS in the treatment of CPID. The metabolomics and network pharmacology common pathways were considered the key metabolic pathways, and the key targets were screened from the potential targets obtained by network pharmacology through the proteins in the key pathways. Finally, the mechanism of KFXYS in the treatment of CPID was explored through literature research (Figure 1).

## Materials and Methods

### Chemicals and Reagents

Kangfuxiaoyan suppository (batch No. 20171225) was obtained from the Sunflower Pharmaceutical Group Co., Ltd. Sodium pentobarbital (batch No. 11011) was obtained from Merck. Mass spectrometry-grade acetonitrile and methanol, and HPLC-grade formic acid were obtained from Thermo Fisher Scientific. The water used for LC/MS analysis was purchased from Watsons.

### Animal Model Establishment and Treatment

**Animal** Sprague Dawley (SD) rats, female, weight 190–210 g, SPF grade, approval number: SCXK (Beijing) 2016-0011, raised in the Animal Experimental Center of Beijing University of Chinese Medicine, the temperature was controlled at 21–25°C, the relative humidity was 55-65%, and the cycle of day and night was 12 h alternately. The rats adapted to the environment for 7 days, during which they drank and ate freely.

**Animal Model Establishment** The rat model of CPID was established by implanting extraneou materials. The method of establishing CPID model is shown in Supplementary Material. The rats are grouped as follows: 20 rats were randomly divided into the normal group and the sham operation group, the normal group did not do any treatment, the sham operation rats received only a 2 cm incision wound in the middle of the lower abdomen, and then sutured without any treatment, and the other rats were used to establish the rat model of CPID by implanting extraneous materials. Finally, the rats were divided into normal group (*n* = 10), sham operation group (*n* = 8), model group (*n* = 10), KFXYS-dosed group (*n* = 11), levofloxacin-dosed group (*n* = 10).

Appropriate amounts of KFXYS and normal saline were added into the clean evaporation dish, heat in a water bath at 50°C until melting, and finally, obtain 0.4 g mL^−1^ KFXYS solution. KFXYS was given by rectal administration according to the clinical equivalent dose of 408 mg kg^−1^ for 21 days, and the rats in each group ate normal diet during the treatment.

### Sample Collection and Preparation

**Sample Collection** For the identification of ingredients absorbed into blood of KFXYS and the study of pharmacokinetic, the blood was collected from orbital vein at different time points (15, 30 min, 1, 2, 3, 4, 6, 8, 12, and 24 h) after the last administration of KFXYS in CPID rats, and placed in a centrifuge tube covered with heparin sodium, centrifuged at 1,100 ×*g* for 10 min at 4°C, and the plasma samples were collected.

For the study of metabolomics, the blood samples were collected from the abdominal aorta in each group rats, and these samples were placed at room temperature for half an hour, centrifuged at 1,100 ×g for 10 min at 4°C, then the serum samples were collected, including eight sham operation group serum samples, 10 model group serum samples, and 11 KFXYS-dosed group serum samples.

**Sample Preparation** For the identification of ingredients absorbed into blood of KFXYS, mixed the plasma sample from each time point evenly, took 1 ml mixed plasma sample, three times the amount of methanol was added to precipitate the protein, and vortex for 30 s, centrifuged at 13,600 ×g for 15 min at 4°C, and the supernatant was dried with nitrogen. Then the residue was redissolved with 100 μL 70% methanol, centrifuged at 13,600 ×g for 10 min at 4°C, and the supernatant was taken as the plasma sample to be tested in each group. As to the study of pharmacokinetic, the plasma samples at each time point were prepared according to the above method.

For the study of metabolomics, in order to obtain comprehensive information of serum metabolites, the polar part and weak polar part of serum samples were treated respectively. The polar samples were processed as follows: taken 120 μL of the serum sample, added 360 μL methanol to precipitate protein, and vortex for 30 s, static 10 min, centrifuged at 13,600 ×*g* for 10 min at 4°C. After the 300 μL supernatant was dried with nitrogen, redissolved with 100 μL acetonitrile-water (1:1, v/v), vortex for 30 s, centrifuged at 13,600 ×*g* for 10 min at 4 °C, and the supernatant was collected. The weak polar samples were processed as follows: taken the serum sample 120 μL, added 300 μL chloroform-methanol (2:1, v/v), vortex for 2 min, static 10 min, centrifuged at 13,600 ×*g* for 10 min at 4°C, then taken 200 μL lower liquid (chloroform layer), dried with nitrogen, redissolved with 100 μL isopropanol-acetonitrile (1:1, v/v), vortex for 30 s, centrifuged at 13,600 ×*g* for 10 min at 4°C, and the supernatant was collected.

### UPLC-Q-TOF/MS Analysis

For the identification of ingredients absorbed into blood of KFXYS, the UPLC-Q-TOF/MS analysis was performed on Waters ACQUITY UPLC I-CLASS liquid phase system equipped with SYNAPT G2-SI high-resolution mass spectrometer. Chromatographic separations were performed at 40°C on ACQUITY UPLC BEH C18 column (2.1 × 100 mm, 1.7 μm, Waters, UK). The mobile phase composed of 0.1% formic acid-water (A) and acetonitrile (B). The elution program was as follows: 0–2 min, 2%–5% B; 2–3.5 min, 5%–8% B; 3.5–6 min, 8%–11% B; 6–8.5 min, 11%–15% B; 8.5–9.5 min, 15%–17% B; 9.5–11 min, 17% B; 11–13.5 min, 17%–20% B; 13.5–15 min, 20%–23% B; 15–17 min, 23%–30% B; 17–20.5 min, 30%–40% B; 20.5–25 min, 40%–60% B; 25–29 min, 60% B; 29–32 min, 60%–100% B; 32–32.1 min, 100%–2% B; 32.1–35 min, 2% B. The flow rate was set to 0.25 ml min^−1^ and the injection volume was 2 μL.

An electrospray ionization source (ESI) was used both in positive and negative mode. The MS conditions were as follows: capillary voltage, +3 KV/-2.5 KV; cone voltage, 40 kV; ion source temperature, 100°C; cone gas flow, 50 L h^−1^; desolvation gas temperature, 400°C; desolvation gas flow, 600 L h^−1^. The Lockmass solution was Leucine encephalin. The collision energy was set as 6 eV (trap) for low-energy scan, and 10–65 eV ramp (trap) for high-energy scan. The mass range was 100-1,200 m*/z* and the data acquisition mode was 3D data acquisition in Continuum mode.

As to the study of pharmacokinetics of main ingredients, the UPLC-Q-TOF/MS analysis was performed according to the above method, but the data acquisition mode was MRM mode.

For the study of metabolomics, all serum samples were performed on Waters Acquity^TM^ UPLC analysis system equipped with Xevo^TM^ G2 Q/TOF tandem quadrupole time-of-flight mass spectrometer, and chromatographic separations were performed at 45°C on ACQUITY UPLC BEH C18 column (2.1 × 100 mm, 1.7 μm, Waters, UK). The UPLC-Q-TOF/MS analysis conditions are shown in Supplementary Material. Quality control (QC) samples were prepared by mixing an equal amount of each sample, and the stability and repeatability of sample analysis were monitored by analyzing QC samples after every 10 samples.

### Identification of Ingredients Absorbed Into Blood of KFXYS

Based on the previous research, the identification steps of the ingredients absorbed into blood of KFXYS were as follows: 1) Summarizing the possible metabolic pathways of various chemical components *in vivo* by comparing with some representative ingredients and tracking the literature. 2) The prototype components and metabolites in the KFXYS-dosed plasma were discovered by comparing with the KFXYS-treated and KFXYS sample. Then the ingredients absorbed into blood of KFXYS were comprehensively identified based on the mass fragmentation pattern, characteristic fragments and chromatographic retention behavior.

### Study on the Pharmacokinetics of Main Components Absorbed into Blood of KFXYS

The optimized ion pairs of the main components and parameters of cone voltage and capillary voltage are shown in Table 1.

**TABLE 1:** Mass spectrum parameters of the main components in MRM mode.

References substance	Parent ions	Ion pairs	Cone voltage (V)	Capillary voltage (V)
Matrine	[M + H]^+^	249.221→148.086	56	26
		249.221→97.945	56	32
Sophocarpine	[M + H]^+^	247.21→136.05	60	24
		247.21→95.99	60	32
Sophoridine	[M + H]^+^	249.22→97.9	70	24
		249.22→83.99	70	30
Oxymatrine	[M + H]^+^	265.22→205.18	62	22
		265.22→97.96	62	28
Oxysophocarpine	[M + H]^+^	263.14→136.16	56	26
		263.14→96.01	56	30

### Metabolomics Data Analysis

The raw files were imported into Progenesis QI software for denoising, peak identification, peak alignment, normalization and other operations. The raw data were converted into a data matrix containing *t*_*R*_-*m/z* ion pairs, sample names and peak intensities, and then the matrix was imported into multivariate statistical software EZinfo2.0 (Waters Corporation, Milford, MA, United States) for principal components analysis (PCA) and orthogonal partial least-squares discriminant analysis (OPLS-DA). The metabolites were identified by searching and comparing the mass fragment information and accurate mass number with data from METLIN (http://metlin.scripps.edu/), HMDB (http://www.hmdb.ca/) and KEGG (http://www.genome.jp/kegg/) databases. The MetaboAnalyst (https://www.metaboanalyst.ca/) was used for metabolic pathway analysis.

### Targets Prediction and Enrichment Analysis

The potential targets of components absorbed into blood of KFXYS were obtained from PharmMapper (http://www.lilab-ecust.cn/pharmmapper/) and TCMSP (https://old.tcmsp-e.com/tcmsp.php) databases. The disease targets related to CPID were searched from OMIM (https://omim.org/) and GeneCards (https://www.genecards.org/) databases. The target protein names were corrected to official gene names by Uniprot (https://www.uniprot.org/). Through the Venn diagram, the components targets and the disease targets intersected, and the intersectional targets were considered potential therapeutic targets of KFXYS in CPID. R-packet ClusterProfiler was used to analyze the potential targets for GO and KEGG enrichment analysis.

### Integration Analysis of Network Pharmacology and Metabolomics

By comparing the potential pathways enriched by the network pharmacological targets with the metabolic pathways enriched by differential metabolites, the common metabolic pathways which were regarded as the key pathways were screened out. The KEGG database was used to obtain the proteins contained in the key metabolic pathways to screen the key targets from the potential targets of network pharmacology. Through the literature research to reveal the possible mechanism of KFXYS in the treatment of CPID.

## Results

### Reversal Effect of KFXYS on Inflammatory Indexes in CPID Rats

**FIGURE 2:**
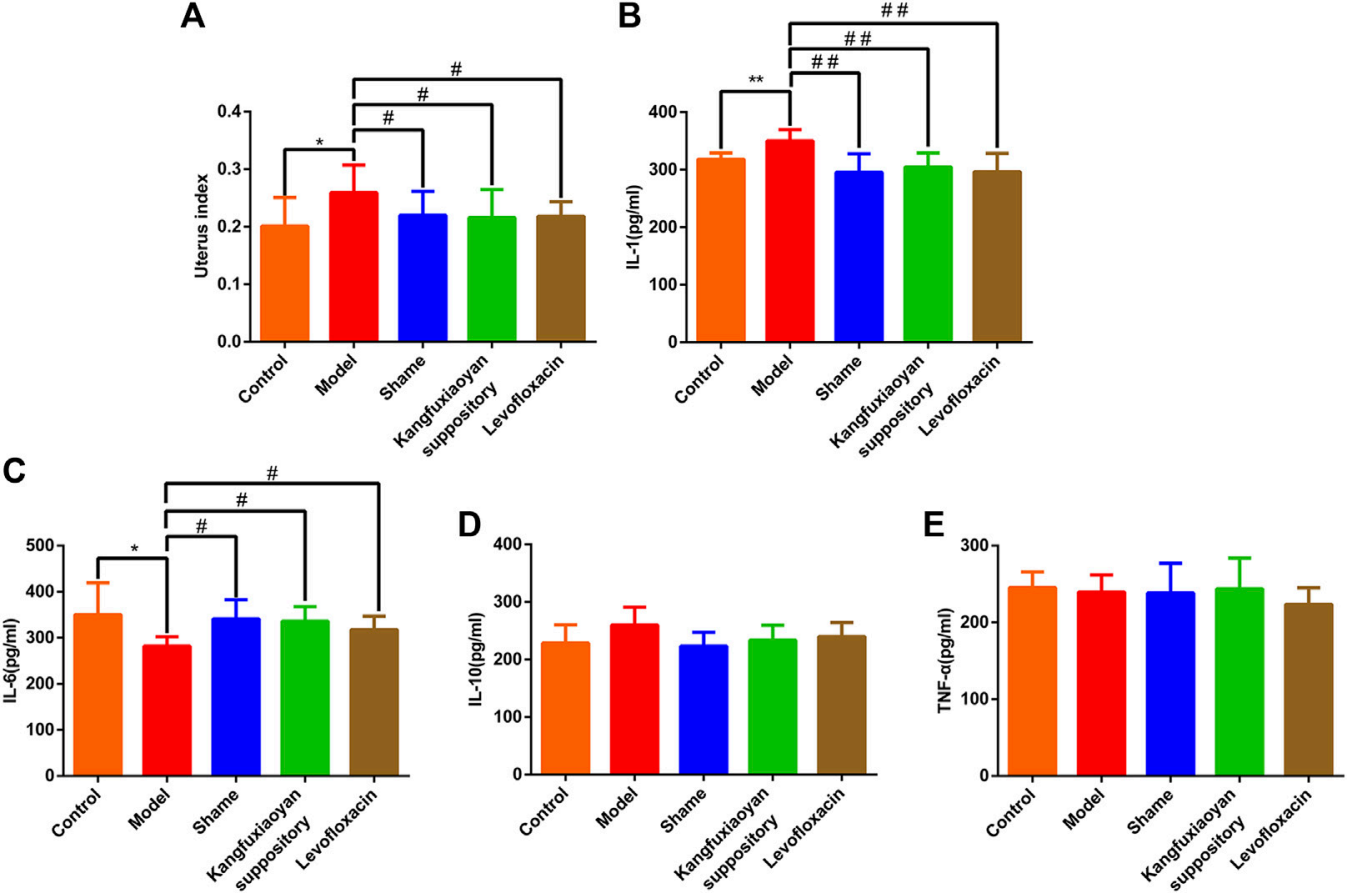
Reversal effect of KFXYS on inflammatory indexes. Effect of KFXYS on **(A)** uterus index, the level of **(B)** IL-1 **(C)** IL-6 **(D)** IL-10 **(E)** TNF-α of rats with CPID. Data are presented as mean ± SD. ^*^ *p* < 0.05, ^**^ *p* < 0.01 vs control group, ^#^ *p* < 0.05, ^##^ *p* < 0.01 vs model group.

In this study, in order to determine whether KFXYS reverse inflammatory indexes to exert anti-inflammatory effect, we measured the level of IL-1, IL-6, IL-10, TNF-α in the uterine tissue homogenate. As shown in Figure 2A, compared with the sham operation group, the uterus index of the model group was significantly increased (*p* < 0.05), and the results indicated that the CPID model was successfully established. Compared with the model group, the uterus index decreased significantly after administration of KFXYS (*p* < 0.05), indicating that KFXYS can effectively improve the degree of uterine swelling in CPID model rats. In the uterine tissue homogenate, the level of IL-6 in the model group decreased significantly (*p* < 0.05), and the level of IL-1 increased significantly (*p* < 0.01). After administration of KFXYS, the changes of these indicators were significantly improved. The result shows that KFXYS has the effect of reversing inflammatory indexes (Figures 2B–E).

## Study of the Ingredients of KFXYS Absorbed Into Blood

### Identification of the Ingredients Absorbed Into Blood of KFXYS

Eighty chemical components with high response and 43 chemical components with weak response from KFXYS were identified by high-resolution mass spectrometry (Supplementary Figure S1), and the components information is shown in Supplementary Table S1.

**FIGURE 3:**
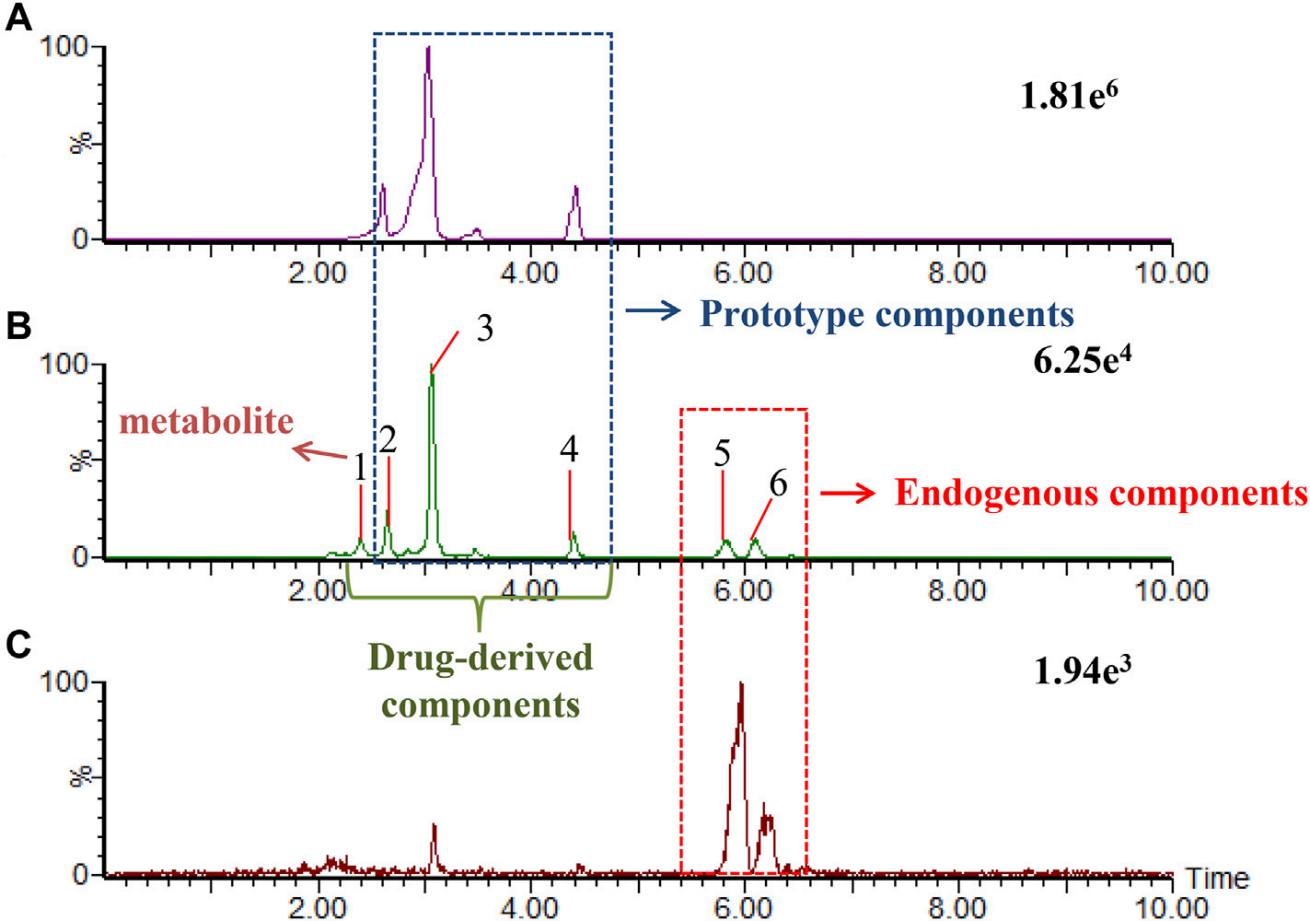
The extraction ion chromatogram at m/z 247.1812 in positive ion mode, which were KFXYS sample **(A)**, KFXYS-dosed plasma sample **(B)**, and blank plasma sample **(C)**.

We developed a new method of real-time binary comparison to discover the ingredients of KFXYS absorbed into the blood (Figure 3), a total of 60 KFXYS-derived compounds were identified, including 10 prototype compounds (Table 2) and 50 metabolites (Supplementary Table S2). According to the metabolic rules established by representative reference substances, we consider that these 60 ingredients were mainly derived from SFR, AH, TH and VH, mainly including alkaloids, flavonoids and organic acids etc.

**TABLE 2:** Prototype components of KFXYS in blood.

NO.	Identification	Formula	*t*_R_ (min)	[M + H]^+^/[M-H]^-^		Error (ppm)	Response	MS/MS	Type
				Meas. (*m/z*)	Pred. (*m/z*)				
1	*N*-Methylcytisine	C_12_H_16_N_2_O	1.43	205.1331	205.1341	−4.87	3,023	118.0649 [M + H-C_3_H_9_N-CO]^+^	P
2	5-Hydroxysparteine	C_15_H_24_N_2_O_2_	1.87	265.1902	265.1916	−5.28	1,580	164.1094 [M + H-2H-C_6_H_13_N]^+^146 [M + H-2H-H_2_O-C_6_H_13_N]^+^	P
3	Hydroxymatrine	C_15_H_24_N_2_O_2_	2.21	265.1901	265.1916	−5.66	3,774	247.1674 [M + H-H_2_O]^+^150.1226 [M + H-C_5_H_7_NO]^+^148.1125 [M + H-C_5_H_9_NO]^+^	P
4	Matrine	C_15_H_24_N_2_O	2.84	249.1957	249.1967	−4.01	93,503	247.1812 [M + H-2H]^+^148.1125 [M + H-2H-C_5_H_7_NO]^+^150.1299 [M + H-C_5_H_7_NO]^+^120.0802 [M + H-2H-C_5_H_7_NO-CO]^+^	P
5	Sophocarpine	C_15_H_22_N_2_O	3.07	247.1794	247.1810	−6.47	14,091	245.1671 [M + H-2H]^+^179.1531 [M + H-C_4_H_4_O]^+^136.1127 [M + H-2H-C_6_H_7_NO]^+^148.1125 [M + H-2H-C_5_H_7_NO]^+^150.1299 [M + H-C_5_H_7_NO]^+^	P
6	Isomatrine	C_15_H_24_N_2_O	3.21	249.1955	249.1967	−4.82	3,697	247.1812 [M + H-2H]^+^120.0802 [M + H-2H-C_5_H_7_NO-CO]^+^	P
7	13,14-Dehydrolupanine	C_15_H_22_N_2_O	3.47	247.1796	247.1810	−5.66	782	112.0773 [M + H-C_9_H_13_N]^+^	P
8	5,6-Dehydrolupanine	C_15_H_22_N_2_O	4.40	247.1794	247.1810	−6.47	2044	148.1125 [M + H-C_6_H_13_N]^+^120.0802 (65) [M + H-C_6_H_13_N-CO]^+^	P
9	Esculetin	C_9_H_6_O_4_	6.78	177.0190	177.0188	1.13	1,683	133.0273 [M-H-CO_2_]^-^	P
10	10-Hydroxyaloin	C_21_H_22_O_10_	17.15	433.1135	433.1135	0.00	2,204	270.0536 [M-H-C_6_H_11_O_5_]^-^253.0472 [M-H-C_6_H_11_O_5-_OH]^-^253.0404 [M-H-C_6_H_11_O_5_-H_2_O]^-^	P

### Study on the Pharmacokinetics of Main Components Absorbed Into Blood of KFXYS

**FIGURE 4:**
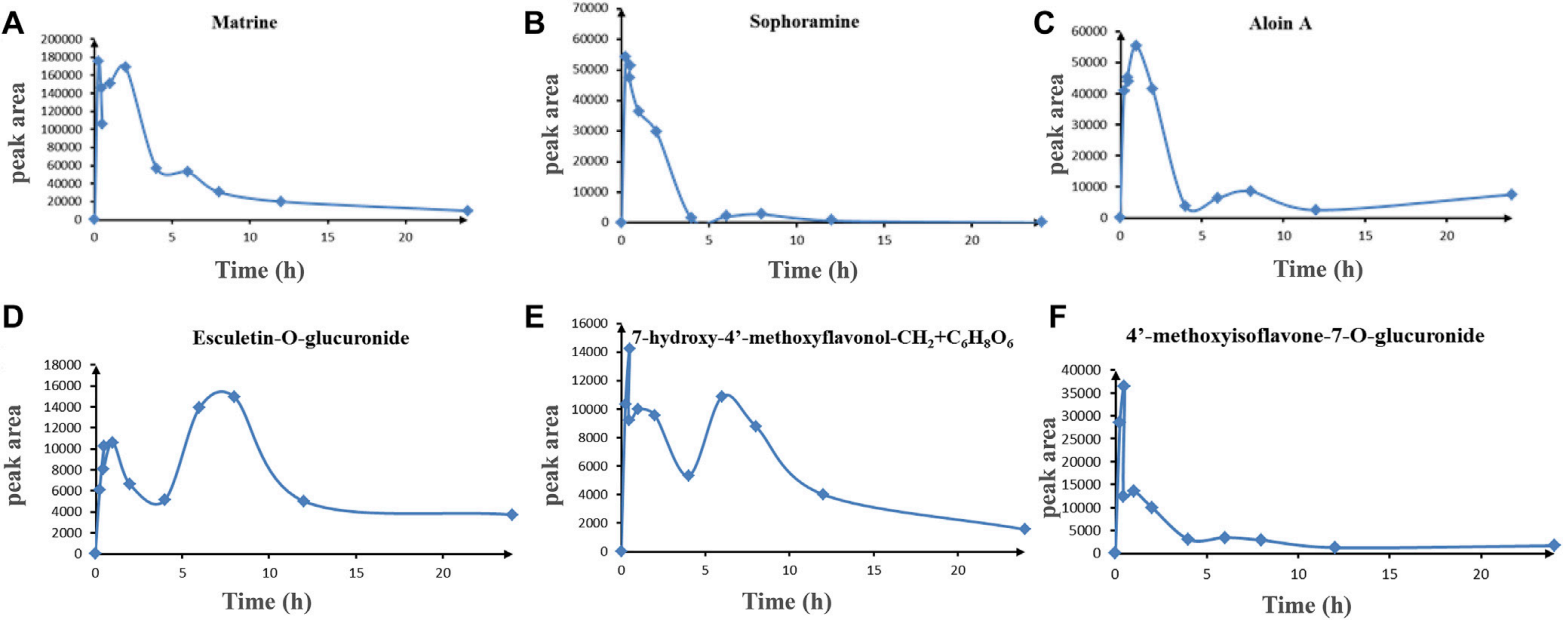
The ADME behavior of transitional components in blood. **(A)** Matrine, **(B)** Sophocarpine, **(C)** Aloin A, **(D)** Esculetin-*O*-glucuronide, **(E)** 7-hydroxy-4′-methoxyflavonol-CH_2_+ C_6_H_8_O_6_, **(F)** 4′-methoxyisoflavone-7-*O*-glucuronide.

In order to explore the drug-like properties of compounds, we investigated the pharmacokinetics of the KFXYS-derived components with good peak shape and considerable content in the blood. It is reported that the alkaloid in SFR has definite anti-inflammatory activity, such as matrine (Pu., et al., 2016), oxymatrine (Wang et al., 2015), sophocarpine (Jiang et al., 2018b); sophoridine (Wang et al., 2021), and oxysophocarpine (Yang et al., 2015). Therefore, we studied the pharmacokinetics of the alkaloids from SFR, including matrine, sophocarpine, sophoridine, oxymatrine and oxysophorine, formononetin from SFR, aloin A from aloe, and esculetin from VH and TH. The results showed that matrine, sophocarpine, aloin, esculetin-*O*-glucuronide, 7,4′-dihydroxyisoflavone-*O*-glucuronide (7-hydroxy-4′-methoxyflavonol-CH_2_+C_6_H_8_O_6_), and 4′-methoxyisoflavone-7-*O*-glucuronide had good ADME behavior *in vivo* (Figure 4). Due to the low content of other components in blood, we only discovered these six components showed good ADME behavior *in vivo*. Thus, we speculated that matrine, sophocarpine, aloin, esculetin, and 7-hydroxy-4′-methoxyisoflavone and their metabolites may be the potential pharmacological components of KFXYS.

## Study on the Mechanism of KFXYS in the Treatment of CPID

### Study of Differential Metabolites Associated With the Inflammatory Indexes of KFXYS

By dividing the serum sample into polar part and weak polar part, more comprehensive metabolic information was obtained, and the BPIs in positive and negative ion mode are shown in Supplementary Figure S2. QC samples in positive and negative ion mode showed that both the instrument precision and method precision can carry out good metabolic profile analysis.

**FIGURE 5:**
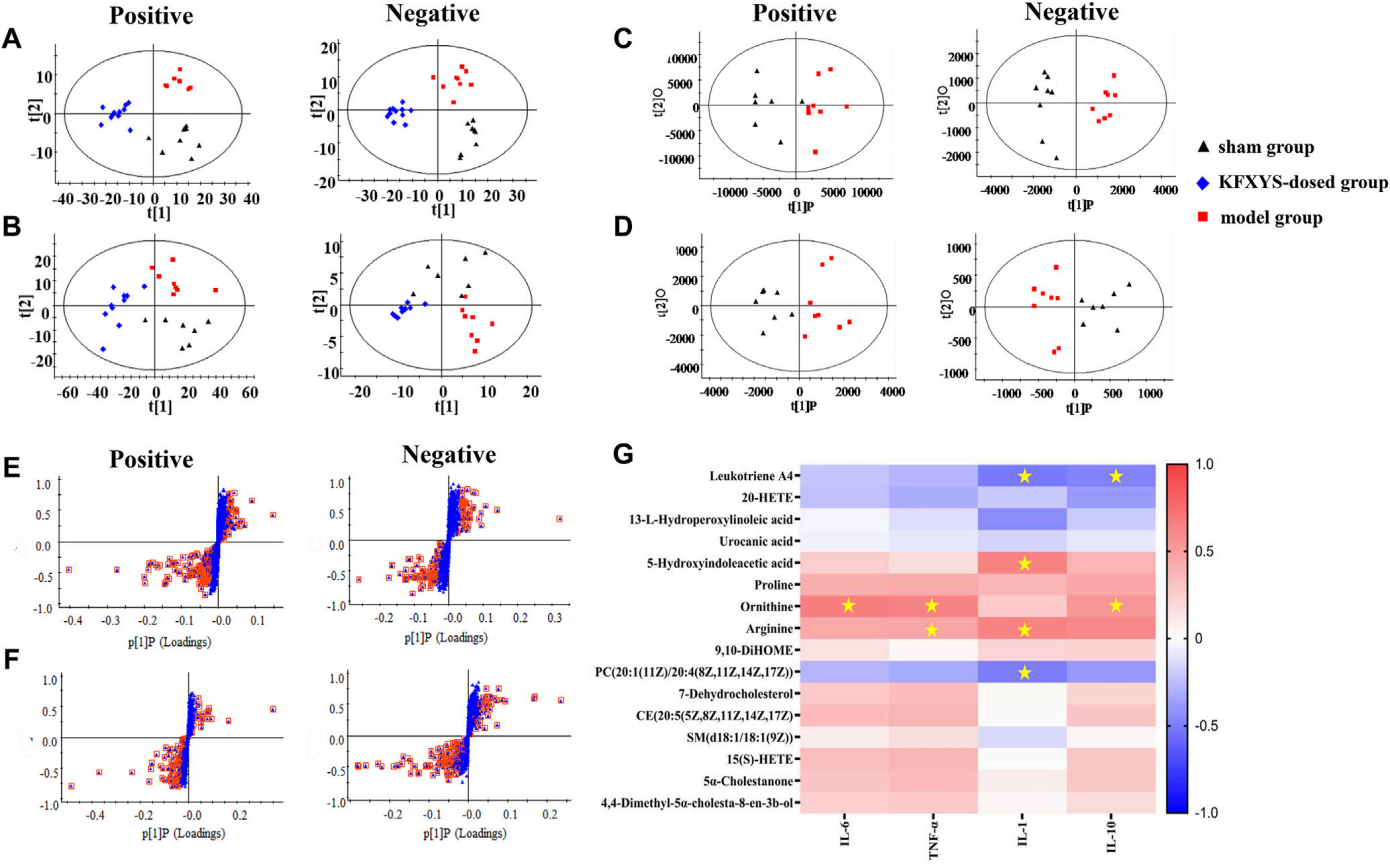
Metabolomics analysis of KFXYS in rats with CPID. PCA analysis in different groups of the polar parts **(A)** and weak polar parts of serum **(B)**. OPLS-DA score plot between the sham operation group and the model group of the polar parts of serum **(C)** and weak polar parts of serum **(D)**; S-plot analysis of rats between the sham operation and the model group of the polar parts of serum **(E)** and weak polar parts of serum **(F)**. **(G)** Pearson correlation between 16 differential metabolites and four inflammatory indexes. The scale ranged from +1.0 (red) to -1.0 (blue). ^*^ *p* < 0.05 denoted statistical significance between metabolites and inflammatory indexes.

The serum samples of sham operation group, model group and KFXYS-dosed group were subject to multivariate statistical analysis. In order to distinguish and visualize the differences between different groups, PCA and OPLS-DA analyses were performed. PCA diagrams showed that the model group and sham operation group are clearly separated, and the KFXYS-dosed group tends to be adjusted to the sham operation group **(** Figures 5A,B). The serum samples of sham operation group and model group were used for Student’s *t*-test (*t*-test) and OPLS-DA analysis (Figures 5C,D) to screen the metabolites with significant differences (*p* < 0.05, VIP >1) (Figures 5E,F), respectively. Among these differential metabolites (*p* < 0.05, VIP > 1), those with the opposite trend in the KFXYS group and model group were defined as final differential metabolites. As shown in Table 3, a total of 16 final differential metabolites were screened and identified.

**TABLE 3:** Differential metabolites of metabolomics.

No	*t*_R_-*m/z*	Quasi-molecular ion	Formula	Name	VIP	T^M^	T^K^
1	0.90–301.2170	M-H_2_O + H	C_20_H_30_O_3_	Leukotriene A4	1.50	↓^*^	↑^**^
2	1.38–301.2166	M-H_2_O-H	C_20_H_32_O_3_	20-HETE	1.63	↑^***^	↓^*^
3	1.81–311.222	M-H	C_18_H_32_O_4_	13-l-Hydroperoxylinoleic acid	3.00	↓^**^	↑
4	3.47–139.0513	M + H	C_6_H_6_N_2_O_2_	Urocanic acid	1.86	↓^**^	↓
5	5.42–192.0663	M + H	C_10_H_9_NO_3_	5-Hydroxyindoleacetic acid	1.17	↑^**^	↓^**^
6	7.69–116.0717	M + H	C_5_H_9_NO_2_	Proline	1.77	↓^**^	↑
7	7.84–133.0983	M + H	C_5_H_12_N_2_O_2_	Ornithine	1.47	↓^*^	↑^*^
8	8.68–173.1038	M-H	C_6_H_14_N_4_O_2_	Arginine	2.25	↑^**^	↓^*^
9	1.6–313.2378	M-H	C_18_H_34_O_4_	9,10-DiHOME	1.86	↑^**^	↓^**^
10	13.03–836.6166	M + H	C_48_H_86_NO_8_P	PC(20:1 (11Z)/20:4 (8Z,11Z,14Z,17Z))	4.93	↓^*^	↑^*^
11	14.98–367.3363	M-H_2_O + H	C_27_H_44_O	7-Dehydrocholesterol	1.85	↓^*^	↑
12	15.02–671.5767	M + H	C_47_H_74_O_2_	CE (20:5 (5Z,8Z,11Z,14Z,17Z)	3.06	↓^*^	↑^*^
13	15.02–711.569	M-H_2_O + H	C_41_H_81_N_2_O_6_P	SM(d18:1/18:1 (9Z))	2.22	↑^**^	↓
14	15.03–303.2323	M-H_2_O + H	C_20_H_32_O_3_	15(S)-HETE	1.59	↓^*^	↑^*^
15	16.29–369.352	M-H_2_O + H	C_27_H_46_O	5α-Cholestanone	8.23	↓^*^	↑
16	16.69–397.3833	M-H_2_O + H	C_29_H_50_O	4,4-Dimethyl-5*α*-cholesta-8-en-3b-ol	3.14	↓^*^	↑

To investigate the correlation between these metabolites and the efficacy of KFXYS, Pearson correlation analysis was performed using the inflammatory indexes in uterine tissue homogenate including IL-6, IL-10, IL-1, and TNF-α, and 16 differential metabolites to further screen metabolites (Figure 5G). The Pearson correlation analysis revealed that inflammatory indexes were significantly correlation to five metabolites, including Leukotriene A4, 5-Hydroxyindoleacetic acid, Ornithine, Arginine, and PC (20:1 (11Z)/20:4 (8Z,11Z,14Z, 17Z)), which indicated that KFXYS may play a role in the treatment of CPID by regulating these directly related metabolites.

**FIGURE 6:**
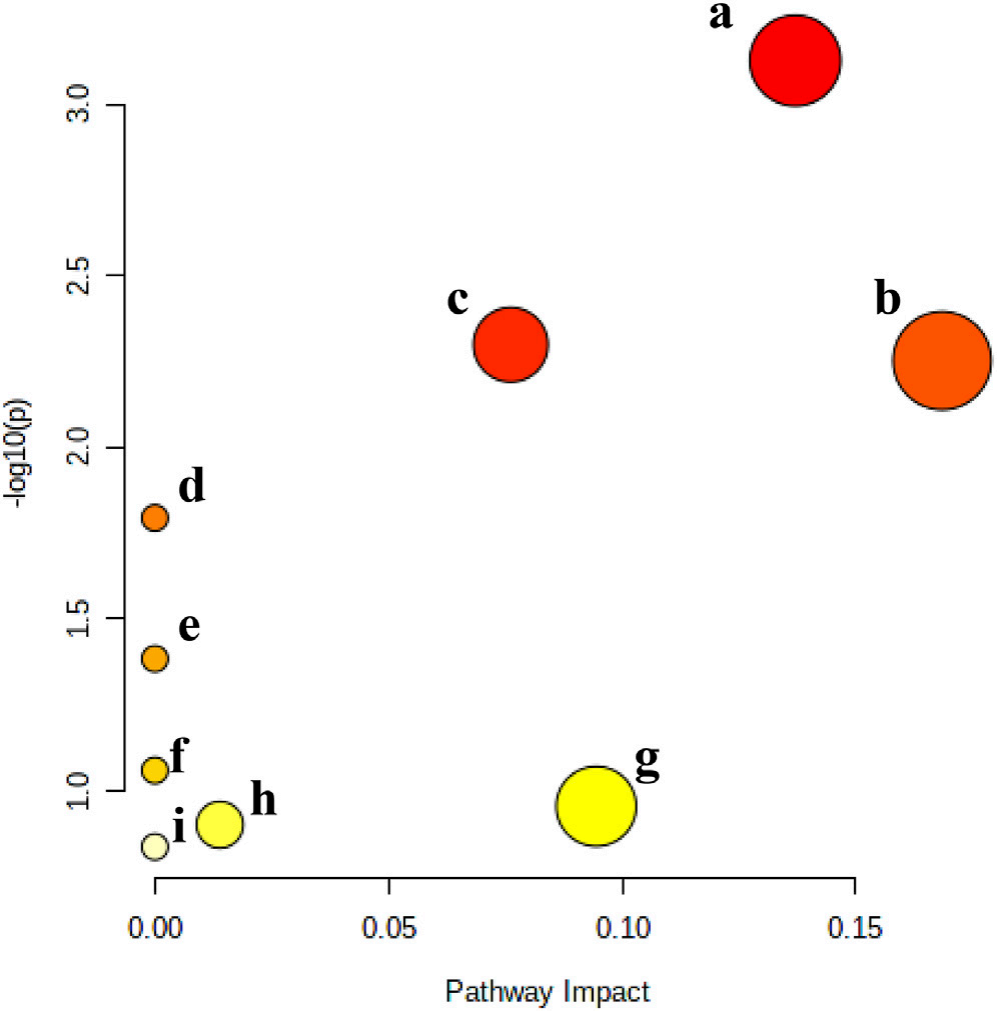
Metabolic pathway analysis of the final differential metabolites. **(A)**. Arginine biosynthesis, **(B)**. Arachidonic acid metabolism, **(C)**. Arginine and proline metabolism, **(D)**. Linoleic acid metabolism, **(E)**. alpha-Linolenic acid metabolism, **(F)**. Glutathione metabolism, **(G)**. Glycerophospholipid metabolism, **(H)**. Tryptophan metabolism, and **(I)**. Aminoacyl-tRNA biosynthesis.

To explore the metabolic pathways related to the efficacy of KFXYS, the five metabolites were imported into MetaboAnalyst. Nine corresponding pathways were constructed, including Arginine biosynthesis, Arachidonic acid metabolism, Arginine and proline metabolism, Linoleic acid metabolism, alpha-Linolenic acid metabolism, Glutathione metabolism, Glycerophospholipid metabolism, Tryptophan metabolism, and Aminoacyl-tRNA biosynthesis (Figure 6).

### Study on the Potential Pathways of KFXYS Based on Network Pharmacology

**FIGURE 7:**
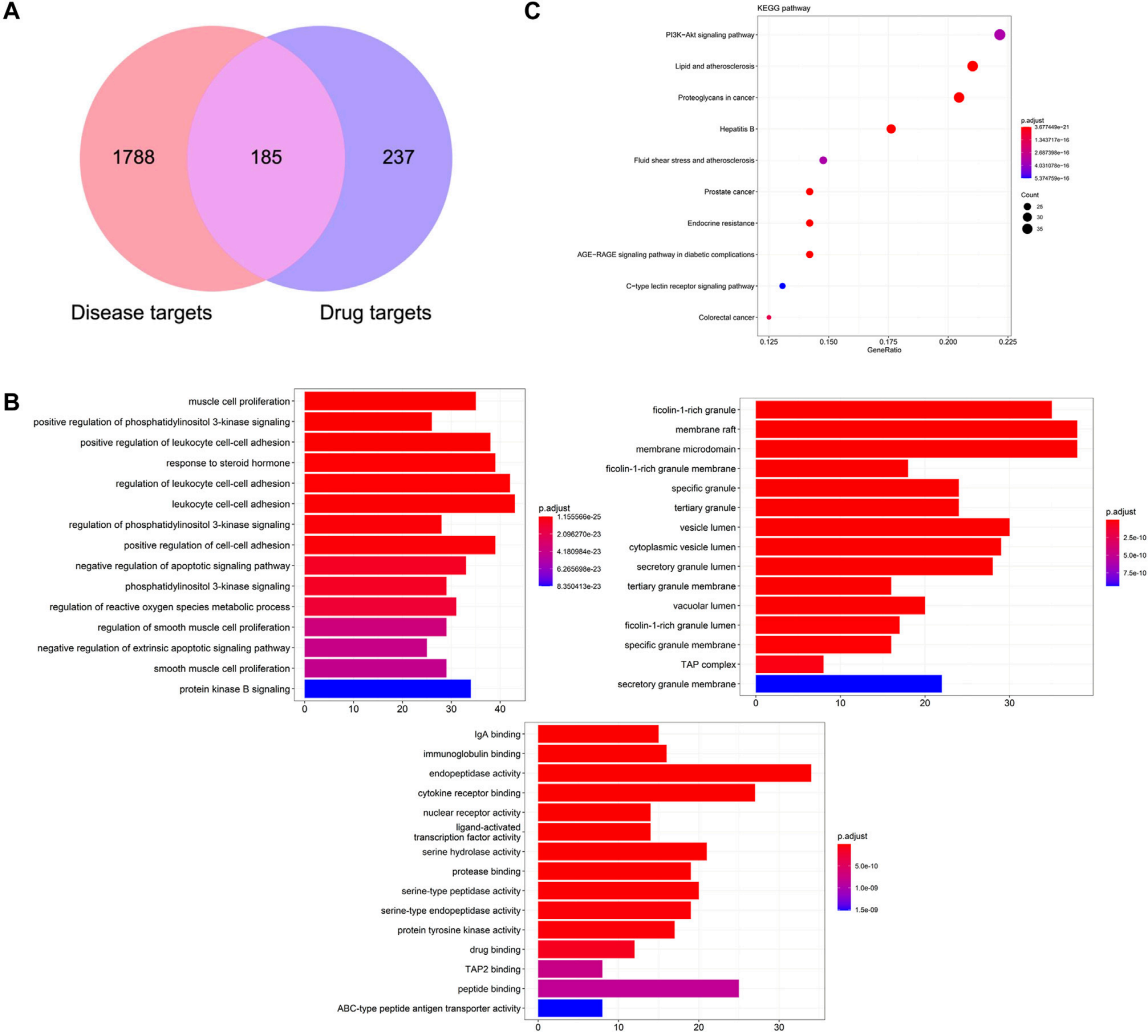
Network pharmacological analysis of KFXYS in the treatment of CPID. **(A)** The overlapped targets of KFXYS and CPID; **(B)** GO terms enrichment analysis (Biological Process, Cellular Component, Molecular Function); **(C)** The KEGG pathway enrichment analysis of the potential targets.

Based on the ingredients absorbed into blood with good ADME behavior, 422 drug targets were predicted from the PharmMapper database and TCMSP database. Combined with GeneCards and OMIM database, 1973 disease targets of CPID were obtained. The 185 overlapping targets of disease targets and drug targets are potential targets for KFXYS in treating CPID (Figure 7A). GO enrichment analysis was used to discover the underlying biological process (BP), cellular component (CC) and molecular function (MF) of the 185 potential targets. BP analysis showed that the treatment of KFXYS was mainly related to response to negative regulation of apoptotic signaling pathway. CC analysis showed that the treatment process of KFXYS was related to cellular components such as membrane raft, membrane microdomain and vesicle lumen. MF analysis showed that the treatment of KFXYS was mainly related to molecular functions such as immunoglobulin binding and cytokine receptor binding (Figure 7B). In KEGG enrichment analysis of potential targets, 176 pathways were obtained with the critical value of *p* < 0.05. The first 10 pathways were taken as shown in Figure 7C.

### Study on the Mechanism of KFXYS Based on the Integration of Metabolomics and Network Pharmacology

**FIGURE 8:**
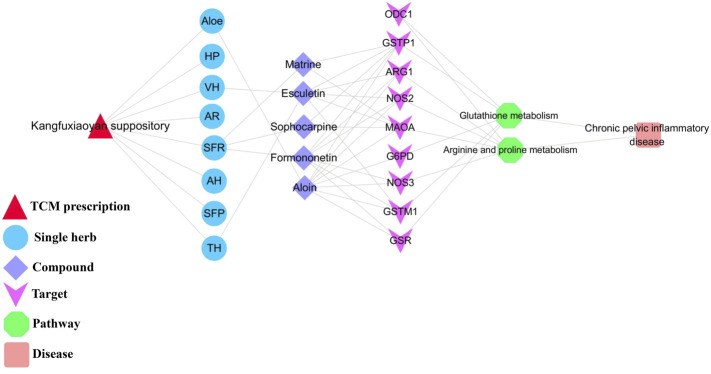
Network diagram of “TCM prescription-single herb-compound-key targets-key pathways-disease".

The shared pathways of metabolomics and network pharmacology were arginine and proline metabolism, glutathione metabolism, which may be the key pathways of KFXYS in the treatment of CPID. The targets obtained by network pharmacology were screened by the proteins in these two key pathways, and the obtained targets were regarded as key targets. Finally, nine key targets were identified. The specific target information is shown in Table 4. According to the previously obtained components absorbed into blood which had good behavior, nine key targets, and two key metabolic pathways, a “TCM prescription-single herb-compound-key targets-key pathways-disease” network of KFXYS in the treatment of CPID was constructed (Figure 8). In this network, it can be seen that KFXYS is characterized by multi-components, multi-targets and multi-pathways in the treatment of diseases. For example, both aloin and formononetin in KFXYS can treat CPID by acting on ARG1, NOS2, and NOS3 proteins in the arginine and proline metabolism.

**TABLE 4:** The key targets of KFXYS in the treatment of CPID.

No	Target	Uniprot id	Protein name
1	NOS3	P29474	Nitric oxide synthase, endothelial
2	NOS2	P35228	Nitric oxide synthase, inducible
3	ARG1	P05089	Arginase-1
4	MAOA	P21397	Amine oxidase
5	ODC1	P11926	Ornithine decarboxylase
6	G6PD	P11413	Glucose-6-phosphate 1-dehydrogenase
7	GSTM1	P09488	Glutathione S-transferase Mu 1
8	GSTP1	P09211	Glutathione S-transferase P
9	GSR	P00390	Glutathione reductase, mitochondrial

## Discussion

TCM is characterized by multi-component, multi-target and multi-pathway for the treatment of diseases. Metabolomics can obtain the changes of endogenous metabolites, and network pharmacology can reveal the potential targets of TCM in the treatment of diseases. The combination of metabolomics and network pharmacology provides a promising strategy for elucidating the treatment mechanisms of TCM. In our study, the reversal effect of KFXYS on inflammatory indexes were verified in CPID rats, and 60 components absorbed into the blood of KFXYS were identified, and among them there are six ingredients had good ADME behavior. Five differential metabolites correlation to inflammatory indexes were discovered by metabolomics and correlation analysis, and these metabolites were used to metabolic pathway analysis. Combined with the potential pathways obtained by network pharmacology, two shared pathways were considered as key pathways, namely, arginine and proline metabolism, glutathione metabolism. The potential targets obtained by network pharmacology were screened with the proteins contained in the key pathways, and nine key targets were screened out.

The components of KFXYS absorbed into the blood and had good ADME behavior may the effective ingredients in treating CPID, and a half of them come from SFR. SFR is the monarch drug of KFXYS, and it is a typical anti-inflammatory drug in TCM (Wang et al., 2015). Among these components, matrine (You et al., 2020) can reduce the levels of pro-inflammatory factors TNF-α, and IL-1β in the disease site to exert anti-inflammatory effect. Sophocarpine (Jiang Z. et al., 2018) can inhibit NF-κB pathway, and up-regulate the activity and expression of endogenous antioxidants such as SOD, CAT and GSH to reduce the level of oxidative stress. Both formononetin (Yi et al., 2020) and aloin (Lee et al., 2019) have effect of anti-inflammatory and antioxidant activity. Esculetin can reduce the secretion of NO and alleviate the damage of inflammation to organs and tissues. A variety of components of KFXYS are working together to exert its therapeutic efficacy.

In arginine and proline metabolism, the level of arginine in serum of rats with CPID increased significantly, while the levels of ornithine and proline decreased significantly. We speculate that the degradation pathway of arginine is inhibited in rats with CPID, which increases the content of arginine and increase the cytotoxicity of specific immune cells (Li et al., 2007). After administration of KFXYS, the levels of ornithine and proline increased, indicating that KFXYS promote the degradation of arginine, and arginase Ⅰ or Ⅱ is the most related to arginine degradation, so it is speculated that KFXYS can increase the expression level of ARG1, promote arginine degradation and improve immune status. Nitric oxide synthase (NOS3, NOS2) can convert l-arginine into citrulline and NO, the excessive production of NO is closely related to mycoplasma-induced infectious inflammation, such as pelvic inflammation (Lin et al., 2021), and NO can cause tissue damage in the process of chronic inflammation (Sioutas et al., 2008). Therefore, the excessive production of NO through downregulate the expression of NOS3 may be an effective strategy for the treatment of inflammation-related diseases such as CPID (Zhang et al., 2018). Ornithine decarboxylase (ODC1) metabolizes l-ornithine to polyamines. Studies have shown that up-regulation of ODC1 can inhibit the secretion of inflammatory cytokines and inhibit ROS-induced apoptosis (Jiang et al., 2018a), indicating that KFXYS may promote the expression of ODC1 so as to inhibit inflammation. Monoamine oxidase A (MAOA) can catalyze the degradation of monoamine and produce H_2_O_2_, a type of ROS (Li et al., 2020), and KFXYS may reduce the expression of MAOA to increase the antioxidant and anti-inflammatory activities (Pallio et al., 2021).

CPID is a chronic inflammation that induces oxidative stress through the production of ROS (Tsuneyama et al., 2002). In glutathione metabolism, NADPH catalyzed by glucose 6-phosphate dehydrogenase (G6PD) plays an important role in the regeneration of glutathione (GSH) (Jain et al., 2020). Glutathione reductase (GSR) can catalyze the reduction of glutathione disulfide (GSSG) to GSH. GSH is an antioxidant that reduces the state of oxidative stress in the body, so G6PD and GSR play an important role in redox regulation (Kim et al., 2019). Glutathione reductase (GSR) is a class of antioxidant enzymes, oxidative stress induced by the release of ROS from inflammatory sites enhances the expression of GST gene (Morceau et al., 2004). Increased expression of glutathione transferase M1 (GSTM1) inhibits apoptosis induced by cytokines and oxidative stress (Zhao et al., 2019). Endogenous glutathione transferase P1 (GSTP1) inhibits the activation of NF-κB (Jones et al., 2016) and alleviates inflammatory response in a variety of experimental models of tissue injury or inflammation (Sánchez-Gómez et al., 2016). KFXYS may alleviate the CPID by acting on the key proteins in glutathione metabolism, promoting the production of glutathione, reducing oxidative stress and apoptosis caused by inflammation.

**FIGURE 9:**
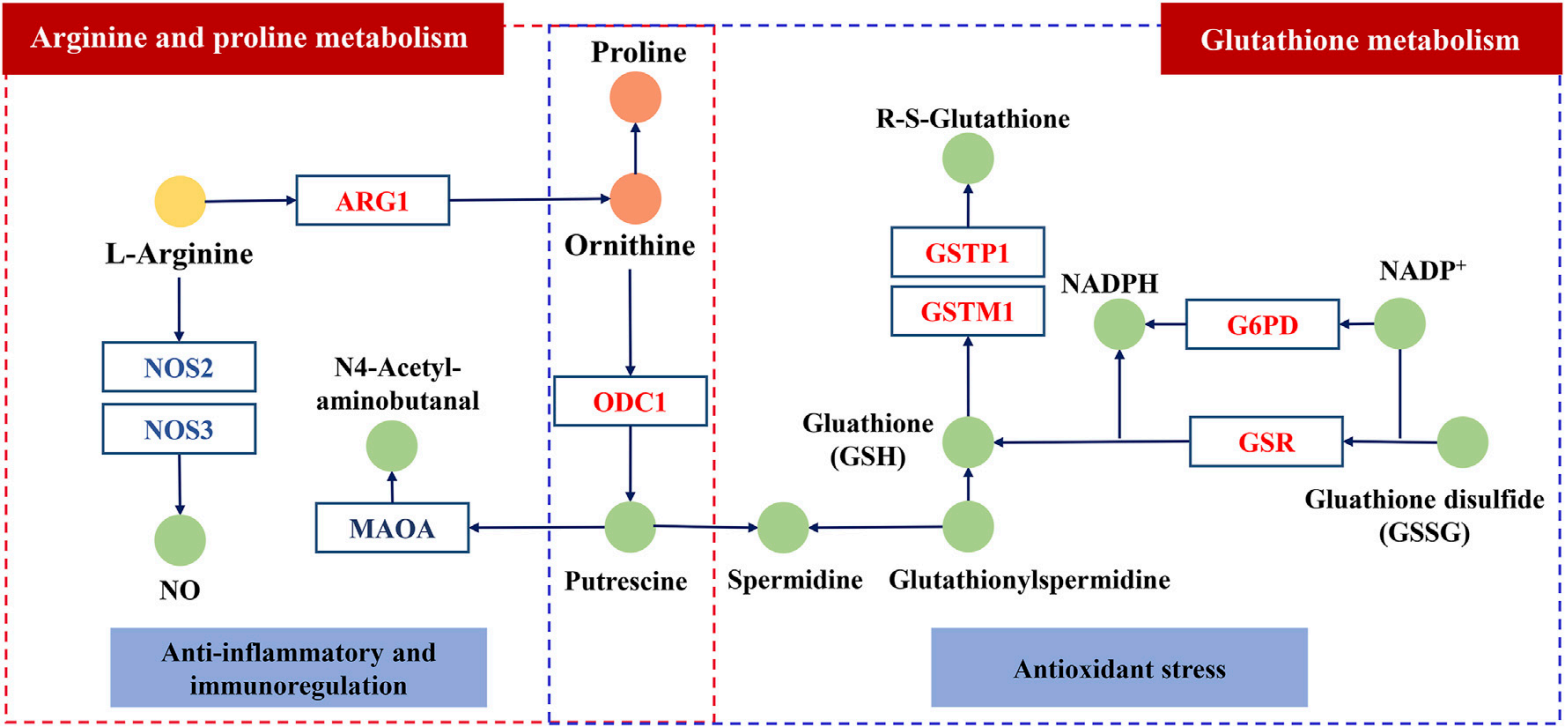
The possible mechanism of KFXYS. The circle represents the metabolite, in which the light yellow circle and the orange circle represent separately the significantly decreased metabolites and the significantly elevated metabolites after administration of KFXYS, and the green circle represents the metabolite that has not been identified by metabolomics. The rectangle represents the key target, which blue font represents inhibition and the red font represents activation.

Overall, we speculate the ingredients of KFXYS may act the key proteins including ARG1, NOS2, NOS3 etc. to play a role in the treatment of CPID (Figure 9). However, the regulatory effect of KFXYS on specific targets needs to be verified by further experiments.

## Data Availability

The original contributions presented in the study are included in the article/Supplementary Material, further inquiries can be directed to the corresponding author.
