# Supplementary material for: Integrated Metabolomics and Network Pharmacology Study on the Mechanism of Kangfuxiaoyan Suppository for Treating Chronic Pelvic Inflammatory Disease
Source: Front Pharmacol. 2022 Feb 4;13:812587. doi: 10.3389/fphar.2022.812587 (PMC8854495; doi:10.3389/fphar.2022.812587)
Supplement: Supplementary file 1 [file DataSheet1.docx]

***Supplementary Material***

Based on high-resolution mass spectrometry, we characterized the components with weak response in KFXYS by increasing detection concentration, optimizing chromatographic separation conditions and online parameters, then the chemical components of KFXYS were comprehensively determined and characterized. As a result, a total 123 chemical components were identified, including 80 chemical components with high response and 43 chemical components with weak response **(Supplementary Figure 1)**, mainly including flavonoids, bile acids, alkaloids etc. The information of the chemical components is shown in **Supplementary Table 1**.

***
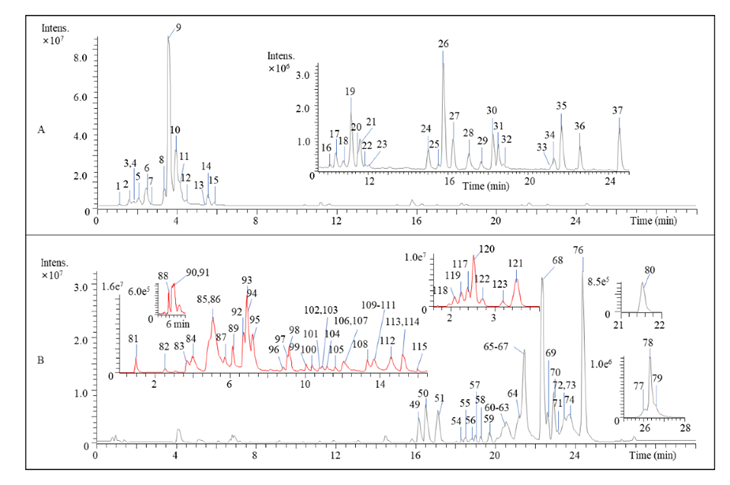
***

**Supplementary Figure 1.** (A) The extracted ion chromatograms of the chemical components from Sophorae Flavescentis Radix (SFR) in the positive ion mode; (B) the BPI chromatograms of the chemical components of KFXYS in the negative ion mode.


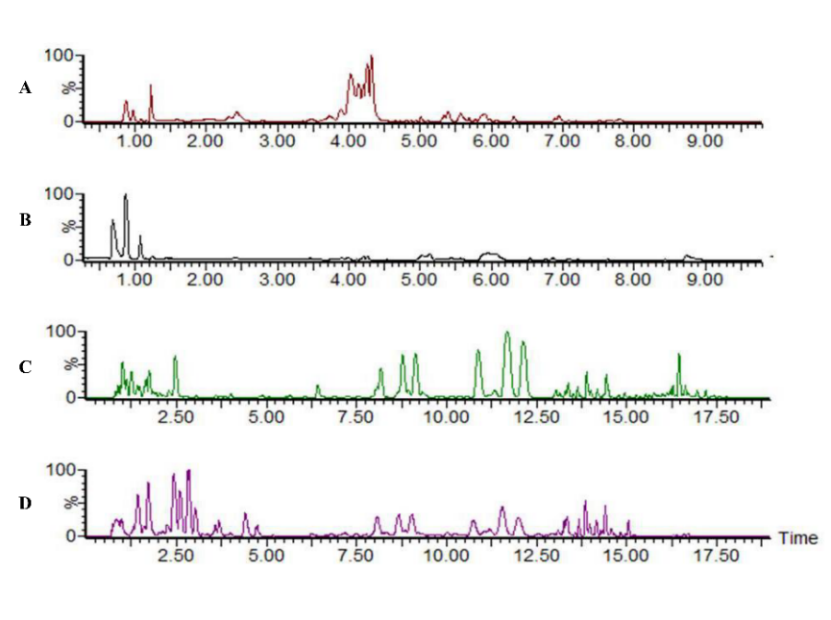


**Supplementary Figure 2** The BPIs of serum samples in positive and negative ion mode. The BPI of polar parts of serum samples in positive ion mode (A) and in negative ion mode(B); The BPI of weak polar parts of serum samples in positive ion mode (C) and in negative ion mode (D).

**Supplementary Tables**

**Supplementary Table 1.** **Table S1 The chemical ingredients of KFXYS**

**Table S1 The** **chemical ingredients of KFXYS**

| **NO.** | **Peak** | ***t*_R_ (min)** | **Identification** | **Formula** | **[M+H]^+^/[M-H]^-^** | | **Error (ppm)** | **MS^2^ (%)** | **MS^3^ (%)** |
| --- | --- | --- | --- | --- | --- | --- | --- | --- | --- |
|  |  |  |  |  | **Pred. (*m/z*)** | **Meas. (*m/z*)** |  |  |  |
| 1 | K1 | 1.03 | N-Methylcytisine or its isomer | C_12_H_16_N_2_O | 205.1335 | 205.1332 | 1.46 | 146.0 (100) [M+H-C_3_H_9_N]^+^  108.0 (75) [M+H-C_6_H_11_N]^+^  110.0 (36) [M+H-C_6_H_9_N]^+^  162.0 (22) [M+H-C_2_H_5_N]^+^ | 118.0 (100) [M+H-C_3_H_9_N-CO]^+^ |
| 2 | K2 | 1.53 | N-Methylcytisine or its isomer | C_12_H_16_N_2_O | 205.1335 | 205.1331 | 1.95 | 146.0 (100) [M+H-C_3_H_9_N]^+^  108.0 (68) [M+H-C_6_H_11_N]^+^  110.0 (30) [M+H-C_6_H_9_N]^+^  162.0 (10) [M+H-C_2_H_5_N]^+^ | / |
| 3 | K3 | 1.65 | Cytisine | C_11_H_14_N_2_O | 191.1178 | 191.1175 | 1.57 | 148.0 (100) [M+H-C_2_H_5_N]^+^ | 120.0 (100) [M+H-C_2_H_5_N-CO]^+^  130 (31) [M+H-C_2_H_5_N-H_2_O]^+^ |
| 4 | K4 | 1.73 | Baptifoline | C_15_H_20_N_2_O_2_ | 261.1597 | 261.1591 | 2.30 | 243.1 (100) [M+H-H_2_O]^+^  114.0 (91) [M+H-C_9_H_9_N]^+^  96.0 (26) [M+H-H_2_O-C_9_H_9_N]^+^ 146.0 (18) [M+H-C_6_H_13_NO]^+^ | 134.0 (100) [M+H-H_2_O-C_6_H_9_N]^+^  96.0 (73) [M+H-H_2_O-C_9_H_9_N]^+^  162.0 (77) [M+H-H_2_O-C_5_H_9_N]^+^  110.0 (43) [M+H-H_2_O-C_9_H_11_N]^+^  215.1 (53) [M+H-H_2_O-CO]^+^ |
| 5 | K5 | 2.03 | 5-Hydroxysparteine | C_15_H_24_N_2_O_2_ | 265.1910 | 265.1901 | 3.39 | 164.0 (100) [M+H-2H-C_6_H_13_N]^+^  247.1 (24) [M+H-H_2_O]^+^  148.0 (16) [M+H-H_2_O-2H-C_5_H_7_NO]^+^  245.1 (18) [M+H-2H-H_2_O]^+^  150.0 (25) [M+H-H_2_O-C_5_H_7_NO]^+^ | 146.0 (100) [M+H-2H-H_2_O-C_6_H_13_N]^+^ 118.0 (30) [M+H-2H-H_2_O-C_6_H_13_N-CO]^+^ |

Table S1 (Continued)

| **NO.** | **Peak** | ***t*_R_ (min)** | **Identification** | **Formula** | **[M+H]^+^/[M-H]^-^** | | **Error (ppm)** | **MS^2^ (%)** | **MS^3^ (%)** |
| --- | --- | --- | --- | --- | --- | --- | --- | --- | --- |
|  |  |  |  |  | **Pred. (*m/z*)** | **Meas. (*m/z*)** |  |  |  |
| 6 | K6 | 2.39 | 9α-Hydroxymatrine | C_15_H_24_N_2_O_2_ | 265.1910 | 265.1900 | 3.77 | 247.1 (100) [M+H-H_2_O]^+^  148.0 (86) [M+H-C_5_H_9_NO]^+^  150.0 (40) [M+H-C_5_H_7_NO]^+^ | 148.0 (100) [M+H-2H-C_5_H_7_NO]^+^  150.0 (66) [M+H-C_5_H_7_NO]^+^ |
| 7 | K7 | 2.46 | Anagyrine or its isomer | C_15_H_20_N_2_O | 245.1648 | 245.1644 | 1.63 | 227.1 (100) [M+H-H_2_O]^+^  228.1 (39) [M+H-OH]^+^  148.0 (24) [M+H-2H-C_6_H_9_N]^+^  162.0 (10) [M+H-C_5_H_9_N]^+^ | 134.0 (28) [M+H-C_6_H_9_NO]^+^  120.0 (14) [M+H-2H-C_6_H_9_N-CO]^+^ |
| 8 | K8 | 2.64 | Anagyrine or its isomer | C_15_H_20_N_2_O | 245.1648 | 245.1644 | 1.63 | 98.0 (100) [M+H-C_9_H_9_NO]^+^  148.0 (45) [M+H-C_7_H_11_N]^+^  227.1 (60) [M+H-H_2_O]^+^ | / |
| 9 | K9 | 3.49 | Isomatrine | C_15_H_24_N_2_O | 249.1961 | 249.1950 | 4.41 | 148.0 (100) [M+H-2H-C_5_H_7_NO]^+^  150.0 (24) [M+H-C_5_H_7_NO]^+^  231.1 (7) [M+H-H_2_O]^+^ | / |
| 10 | K10 | 3.88 | Sophocarpine | C_15_H_22_N_2_O | 247.1805 | 247.1796 | 3.64 | 179.0 (100) [M+H-C_4_H_4_O]^+^ 150.0 (84) [M+H-C_5_H_7_NO]^+^  148.0 (52) [M+H-2H-C_5_H_7_NO]^+^  245.1 (36) [M+H-2H]^+^  136.0 (36) [M+H-2H-C_6_H_7_NO]^+^ | 136.0 (100) [M+H-C_4_H_4_O-2H-C_2_H_3_N]^+^ |

Table S1 (Continued)

| **NO.** | **Peak** | ***t*_R_ (min)** | **Identification** | **Formula** | **[M+H]^+^/[M-H]^-^** | | **Error (ppm)** | **MS^2^ (%)** | **MS^3^ (%)** |
| --- | --- | --- | --- | --- | --- | --- | --- | --- | --- |
|  |  |  |  |  | **Pred. (*m/z*)** | **Meas. (*m/z*)** |  |  |  |
| 11 | K11 | 4.10 | Matrine | C_15_H_24_N_2_O | 249.1961 | 249.1953 | 3.21 | 148.0 (100) [M+H-2H-C_5_H_7_NO]^+^  150.0 (89) [M+H-C_5_H_7_NO]^+^  231.1 (24) [M+H-H_2_O]^+^ | 120.0 (100) [M+H-2H-C_5_H_7_NO-CO]^+^  148.0 (100) [M+H-2H-C_5_H_7_NO]^+^ |
| 12 | K12 | 4.40 | 13,14-Dehydrolupanine | C_15_H_22_N_2_O | 247.1805 | 247.1800 | 2.02 | 112.0 (100) [M+H-C_9_H_13_N]^+^ | 84.0 (100) [M+H-C_9_H_13_N-CO]^+^ |
| 13 | K13 | 5.38 | Sophoramine | C_15_H_20_N_2_O | 245.1648 | 245.1642 | 2.45 | 217.1 (100) [M+H-CO]^+^  227.1 (51) [M+H-H_2_O]^+^  228.0 (10) [M+H-OH]^+^  243.1 (20) [M+H-2H]^+^  148.0 (27) [M+H-C_5_H_5_NO-2H]^+^  150.0 (20) [M+H-C_5_H_5_NO]^+^ | 189.1 (100) [M+H-CO-C_2_H_4_]^+^ |
| 14 | K14 | 5.51 | 5,6-Dehydrolupanine | C_15_H_22_N_2_O | 247.1805 | 247.1798 | 2.83 | 148.0 (100) [M+H-C_6_H_13_N]^+^ | 148.0 (100) [M+H-C_6_H_13_N]^+^  120.0 (65) [M+H-C_6_H_13_N-CO]^+^  131.0 (8) [M+H-C_6_H_13_N-OH]^+^ |
| 15 | K15 | 5.81 | Epilamprolobine | C_15_H_24_N_2_O_2_ | 265.1910 | 265.1902 | 3.02 | 150.0 (100) [M+H-C_5_H_7_NO_2_-2H]^+^ 152.0 (19) [M+H-C_5_H_7_NO_2_]^+^ | 122.0 (100) [M+H-C_5_H_7_NO_2_-2H-C_2_H_4_]^+^ |
| 16 | K16 | 10.10 | 9*α*-Hydroxysophoramine isomer | C_15_H_20_N_2_O_2_ | 261.1597 | 261.1592 | 1.91 | 243.1 (100) [M+H-H_2_O]^+^ | 215.1 (100) [M+H-H_2_O-CO]^+^ |

Table S1 (Continued)

| **NO.** | **Peak** | ***t*_R_ (min)** | **Identification** | **Formula** | **[M+H]^+^/[M-H]^-^** | | **Error (ppm)** | **MS^2^ (%)** | **MS^3^ (%)** |
| --- | --- | --- | --- | --- | --- | --- | --- | --- | --- |
|  |  |  |  |  | **Pred. (*m/z*)** | **Meas. (*m/z*)** |  |  |  |
| 17 | K17 | 10.40 | 3'-Methoxy-4'-hydroxyisoflavone-7-*O*-xylose-(1→6)-glucoside | C_27_H_30_O_14_ | 579.1708 | 579.1691 | 2.94 | 285.1 (100) [M+H-C_11_H_18_O_9_]^+^  447.0 (94) [M+H-C_5_H_8_O_4_]^+^ | 270.0 (100) [M+H-C_11_H_18_O_9_-CH_3_]^+^  253.0 (43) [M+H-C_11_H_18_O_9_-OH]^+^ 225.0 (14) [M+H-C_11_H_18_O_9_-OH-CO]^+^ |
| 18 | K18 | 10.79 | 3'-Methoxy-4'-hydroxyisoflavone-7-*O*-apiose-(1→6)-glucoside | C_27_H_30_O_14_ | 579.1708 | 579.1691 | 2.94 | 285.1 (100) [M+H-C_11_H_18_O_9_]^+^  447.1 (87) [M+H-C_5_H_8_O_4_]^+^ | / |
| 19 | K19 | 11.16 | 5-Hydroxy-4'- methoxyisoflavone-7-*O*-xylose-(1→6)-glucoside | C_27_H_30_O_14_ | 579.1708 | 579.1688 | 3.45 | 285.1 (100) [M+H-C_11_H_18_O_9_]^+^  447.1 (87) [M+H-C_5_H_8_O_4_]^+^ | 270.0 (100) [M+H-C_11_H_18_O_9_-CH_3_]^+^  253.0 (46) [M+H-C_11_H_18_O_9_-OH]^+^  225.1 (17) [M+H-C_11_H_18_O_9_-OH-CO]^+^ |
| 20 | K20 | 11.50 | 5-Hydroxy-4'- methoxyisoflavone-7-*O*-apiose-(1→6)-glucoside | C_27_H_30_O_14_ | 579.1708 | 579.1691 | 2.94 | 285.1 (100) [M+H-C_11_H_18_O_9_]^+^  447.1 (87) [M+H-C_5_H_8_O_4_]^+^ | 270.0 (100) [M+H-C_11_H_18_O_9_-CH_3_]^+^  253.0 (43) [M+H-C_11_H_18_O_9_-OH]^+^  225.0 (16) [M+H-C_11_H_18_O_9_-OH-CO]^+^ |

Table S1 (Continued)

| **NO.** | **Peak** | ***t*_R_ (min)** | **Identification** | **Formula** | **[M+H]^+^/[M-H]^-^** | | **Error (ppm)** | **MS^2^ (%)** | **MS^3^ (%)** |
| --- | --- | --- | --- | --- | --- | --- | --- | --- | --- |
|  |  |  |  |  | **Pred. (*m/z*)** | **Meas. (*m/z*)** |  |  |  |
| 21 | K21 | 11.62 | 2'-Methoxyflavonol-7-*O*-glucoside | C_22_H_22_O_10_ | 447.1286 | 447.1271 | 3.35 | 285.1 (100) [M+H-C_6_H_10_O_5_]^+^ | 269.9 (100) [M+H-C_6_H_10_O_5_-CH_3_]^+^  253.0 (40) [M+H-C_6_H_10_O_5_-CH_3_-OH]^+^ 224.9 (20) [M+H-C_6_H_10_O_5_-CH_3_-OH-CO]^+^ |
| 22 | K22 | 11.60 | 5,4'-Dihydroxyisoflavone-7-*O*-xylose-(1→6)-glucoside | C_26_H_28_O_14_ | 565.1529 | 565.1531 | -0.35 | 433.1 (100) [M+H-C_5_H_8_O_4_]^+^  271.0 (34) [M+H-C_11_H_18_O_9_]^+^ | / |
| 23 | K23 | 12.06 | 5,4'-Dihydroxyisoflavone-7-*O*-apiose-(1→6)- glucoside | C_26_H_28_O_14_ | 565.1530 | 565.1531 | -0.18 | 433.1 (100) [M+H-C_5_H_8_O_4_]^+^  271.0 (34) [M+H-C_11_H_18_O_9_]^+^ | 271.1 (100) [M+H-C_11_H_18_O_9_]^+^ |
| 24 | K24 | 15.00 | Pseudobatigenin-7-*O*-xylose-(1→6)-glucoside | C_27_H_28_O_14_ | 577.1552 | 577.1537 | 2.60 | 445.0 (100) [M+H-C_5_H_8_O_4_]^+^  283.1 (83) [M+H-C_11_H_18_O_9_]^+^ | 577.15-445.0: 283.1 (100) [M+H-C_5_H_8_O_4_-C_6_H_10_O_5_]^+^  577.15-283.1: 253.0 (100) [M+H-C_11_H_18_O_9_-CH_2_O]^+^  225.0 (58) [M+H-C_11_H_18_O_9_-CH_2_O-CO]^+^ |
| 25 | K25 | 15.51 | Pseudobatigenin-7-*O*-apiose-(1→6)-glucoside | C_27_H_28_O_14_ | 577.1552 | 577.1537 | 2.60 | 445.0 (100) [M+H-C_5_H_8_O_4_]^+^  283.1 (66) [M+H-C_11_H_18_O_9_]^+^ | 283.1 (100) [M+H-C_5_H_8_O_4_-C_6_H_10_O_5_]^+^ |

Table S1 (Continued)

| **NO.** | **Peak** | ***t*_R_ (min)** | **Identification** | **Formula** | **[M+H]^+^/[M-H]^-^** | | **Error (ppm)** | **MS^2^ (%)** | **MS^3^ (%)** |
| --- | --- | --- | --- | --- | --- | --- | --- | --- | --- |
|  |  |  |  |  | **Pred. (*m/z*)** | **Meas. (*m/z*)** |  |  |  |
| 26 | K26 | 15.74 | 4'-Methoxyisoflavone-7-*O*-apiose-(1→6)-glucoside | C_27_H_30_O_13_ | 563.1759 | 563.174 | 3.37 | 269.0 (100) [M+H-C_11_H_18_O_9_]^+^  431.1 (92) [M+H-C_5_H_8_O_4_]^+^ | 254.0 (100) [M+H-C_11_H_18_O_9_-CH_3_]^+^  237.0 (46) [M+H-C_11_H_18_O_9_-CH_3_-OH]^+^ |
| 27 | K27 | 16.25 | Kushenol O | C_27_H_30_O_13_ | 563.1759 | 563.174 | 3.37 | 269.0 (100) [M+H-C_11_H_18_O_9_]^+^  431.1 (90) [M+H-C_5_H_8_O_4_]^+^ | 254.0 (100) [M+H-C_11_H_18_O_9_-CH_3_]^+^  237.0 (48) [M+H-C_11_H_18_O_9_-CH_3_-OH]^+^ |
| 28 | K28 | 16.99 | Formononetin or its isomer | C_16_H_12_O_4_ | 269.0808 | 269.0802 | 2.23 | 254.0 (100) [M+H-CH_3_]^+^  237.0 (48) [M+H-CH_3_-OH]^+^ | 237.0 (100) [M+H-CH_3_-OH]^+^  226.1 (20) [M+H-CH_3_-CO]^+^ |
| 29 | K29 | 17.64 | 7-Hydroxy-4’-methoxyflavonol its isomer | C_16_H_12_O_5_ | 285.0757 | 285.0754 | 1.05 | 270.0 (100) [M+H-CH_3_]^+^  253.0 (42) [M+H-CH_3_-OH]^+^  225.0 (17) [M+H-CH_3_-OH-CO]^+^ | 136.0 (100) [M+H-C_9_H_8_O_2_-H_2_O]^+^  253.0 (69) [M+H-CH_3_-OH]^+^ |
| 30 | K30 | 18.22 | 7-Hydroxy-4’-methoxyflavonol its isomer | C_16_H_12_O_5_ | 285.0757 | 285.0752 | 1.75 | 270.0 (100) [M+H-CH_3_]^+^  253.0 (41) [M+H-CH_3_-OH]^+^  225.0 (18) [M+H-CH_3_-OH-CO]^+^ | 136.0 (100) [M+H-C_9_H_8_O_2_-H_2_O]^+^  253.0 (49) [M+H-CH_3_-OH]^+^ |
| 31 | K31 | 18.50 | *l*-Maackiai | C_16_H_12_O_5_ | 285.0757 | 285.0749 | 2.81 | 122.9 (100) [M+H-C_9_H_6_O_3_]^+^ | 94.9 (100) [M+H-C_9_H_6_O_3_-CO]^+^ |

Table S1 (Continued)

| **NO.** | **Peak** | ***t*_R_ (min)** | **Identification** | **Formula** | **[M+H]^+^/[M-H]^-^** | | **Error (ppm)** | **MS^2^ (%)** | **MS^3^ (%)** |
| --- | --- | --- | --- | --- | --- | --- | --- | --- | --- |
|  |  |  |  |  | **Pred. (*m/z*)** | **Meas. (*m/z*)** |  |  |  |
| 32 | K32 | 18.72 | Kushenol H | C_26_H_32_O_8_ | 473.2161 | 473.2169 | -1.69 | 337.1 (100) [M+H-C_8_H_8_O_2_]^+^  319.1 (25) [M+H-C_8_H_8_O_2_-H_2_O]^+^  455.2（17）[M+H-H_2_O]^+^ | 319.1 (100) [M+H-C_8_H_8_O_2_-H_2_O]^+^  179.0 (35) [M+H-C_8_H_8_O_2_-C_9_H_18_O_2_]^+^ |
| 33 | K33 | 21.12 | 5-Methoxy-7,2',4'-trihydroxy-8-prenylflavanone | C_21_H_22_O_6_ | 371.1498 | 371.1489 | 2.42 | 235.0 (100) [M+H-C_8_H_8_O_2_]^+^  315.0 (35) [M+H-C_4_H_8_]^+^ | 179.0 (100) [M+H-C_8_H_8_O_2_-C_4_H_8_]^+^ |
| 34 | K34 | 21.26 | Pseudobatigenin | C_16_H_10_O_5_ | 283.0601 | 283.0596 | 1.77 | 253.01 (100) [M+H-CH_2_O]^+^  225.0 (60) [M+H-CH_2_O-CO]^+^ | 225.0 (100) [M+H-CH_2_O-CO]^+^  197.1 (12) [M+H-CH_2_O-2CO]^+^ |
| 35 | K35 | 21.64 | Formononetin or its isomer | C_16_H_12_O_4_ | 269.0808 | 269.0802 | 2.23 | 254.0 (100) [M+H-CH_3_]^+^  237.0 (48) [M+H-CH_3_-OH]^+^  241.0 (29) [M+H-CO]^+^ | 237.0 (100) [M+H-CH_3_-OH]^+^  226.1 (20) [M+H-CH_3_-CO]^+^ |
| 36 | K36 | 22.57 | Xanthohumol | C_21_H_22_O_5_ | 355.154 | 355.1535 | 1.41 | 299.1 (100) [M+H-C_4_H_8_]^+^ | 179.0 (100) [M+H-C_4_H_8_-C_8_H_8_O]^+^ |
| 37 | K37 | 24.55 | Kurarinone | C_26_H_30_O_6_ | 439.2115 | 439.2106 | 2.05 | 303.1 (100) [M+H-C_8_H_8_O_2_]^+^ | 178.9 (100) [M+H-C_8_H_8_O_2_-C_9_H_16_]^+^ |
| 39 | L1 | 6.78 | Aloesin or its isomer | C_19_H_22_O_9_ | 393.1183 | 393.1180 | 0.76 | 273.0 (100) [M-H-C_4_H_8_O_4_]^-^ | 245.0 (100) [M-H-C_4_H_8_O_4_-CO]^-^  231.0 (65) [M-H-C_4_H_8_O_4_-C_2_H_2_O]^-^  203.1 (8) [M-H-C_4_H_8_O_4_-C_2_H_2_O-CO]^-^ |

Table S1 (Continued)

| **NO.** | **Peak** | ***t*_R_ (min)** | **Identification** | **Formula** | **[M+H]^+^/[M-H]^-^** | | **Error (ppm)** | **MS^2^ (%)** | **MS^3^ (%)** |
| --- | --- | --- | --- | --- | --- | --- | --- | --- | --- |
|  |  |  |  |  | **Pred. (*m/z*)** | **Meas. (*m/z*)** |  |  |  |
| 40 | L2 | 7.38 | Aloesol-8-C-glucoside | C_19_H_24_O_9_ | 395.1339 | 395.1336 | 0.76 | 351.1 (100) [M-H-C_2_H_4_O]^-^  275.1 (32) [M-H-C_4_H_8_O_4_]^-^ | 231.0 (100) [M-H-C_2_H_4_O-C_4_H_8_O_4_]^-^ |
| 41 | L3 | 8.20 | 7-*O*-methylaloesin | C_20_H_24_O_9_ | 407.1340 | 407.1336 | 0.98 | 243.0 (100) [M-H-C_6_H_12_O_5_]^-^  365.2 (55) [M-H-C_2_H_2_O]^-^ | / |
| 42 | L4 | 10.30 | Aloesin or its isomer | C_19_H_22_O_9_ | 393.1181 | 393.1180 | 0.25 | 273.0 (100) [M-H-C_4_H_8_O_4_]^-^ | 245.0 (100) [M-H-C_4_H_8_O_4_-CO]^-^  231.0 (81) [M-H-C_4_H_8_O_4_-C_2_H_2_O]^-^  203.0 (10) [M-H-C_4_H_8_O_4_-C_2_H_2_O-CO]^-^ |
| 43 | L5 | 11.08 | Aloesin or its isomer | C_19_H_22_O_9_ | 393.1185 | 393.1180 | 1.27 | 273.0 (100) [M-H-C_4_H_8_O_4_]^-^  231.1 (7) [M-H-C_6_H_10_O_5_]^-^ | 245.0 (100) [M-H-C_4_H_8_O_4_-CO]^-^  231.0 (56) [M-H-C_4_H_8_O_4_-C_2_H_2_O]^-^ |
| 44 | L6 | 11.90 | 10-Hydroxyaloin A/B | C_21_H_22_O_10_ | 433.1135 | 433.1129 | 1.39 | 270.0 (100) [M-H-C_6_H_11_O_5_]^-^ | 253.1 (100) [M-H-C_6_H_11_O_5_-OH]^-^  252.0 (71) [M-H-C_6_H_11_O_5_-H_2_O]^-^  241.0 (17) [M-H-C_6_H_11_O_5_-CHO]^-^ |
| 45 | L7 | 13.10 | 10-Hydroxyaloin A/B | C_21_H_22_O_10_ | 433.1135 | 433.1129 | 1.39 | 270.0 (100) [M-H-C_6_H_11_O_5_]^-^ | 253.0 (100) [M-H-C_6_H_11_O_5_-OH]^-^  252.0 (71) [M-H-C_6_H_11_O_5_-H_2_O]^-^ 241.0 (25) [M-H-C_6_H_11_O_5_-CHO]^-^ |

Table S1 (Continued)

| **NO.** | **Peak** | ***t*_R_ (min)** | **Identification** | **Formula** | **[M+H]^+^/[M-H]^-^** | | **Error (ppm)** | **MS^2^ (%)** | **MS^3^ (%)** |
| --- | --- | --- | --- | --- | --- | --- | --- | --- | --- |
|  |  |  |  |  | **Pred. (*m/z*)** | **Meas. (*m/z*)** |  |  |  |
| 46 | L8 | 13.39 | Aloeresin A | C_28_H_28_O_11_ | 539.1550 | 539.1549 | 0.19 | 375.1 (100) [M-H-C_9_H_8_O_3_]^-^ | 333.1 (100) [M-H-C_9_H_8_O_3_-C_2_H_2_O]^-^  273.1 (69) [M-H-C_9_H_8_O_3_-C_4_H_6_O_3_]^-^  315.2 (52) [M-H-C_9_H_8_O_3_-C_2_H_2_O-H_2_O]^-^  231.1 (24) [M-H-C_9_H_8_O_3_-C_2_H_2_O-C_4_H_6_O_3_]^-^ |
| 47 | L9 | 14.43 | Rabaichromone | C_29_H_32_O_12_ | 571.1809 | 571.1810 | -0.18 | 527.2 (100) [M-H-C_2_H_4_O]^-^  553.2 (32) [M-H-H_2_O]^-^ | 161.0 (100) [M-H-C_2_H_4_O-C_3_H_4_-C_15_H_18_O_8_]^-^  351.2 (48) [M-H-C_2_H_4_O-C_9_H_6_O_3_-CH_3_]^-^  232.2 (16) [M-H-C_2_H_4_O-C_9_H_6_O_3_-C_4_H_8_O_4_-CH_2_]^-^ |
| 48 | L10 | 14.47 | Aloenin B | C_34_H_38_O_17_ | 717.2018 | 717.2025 | -0.98 | 555.2 (100) [M-H-C_6_H_10_O_5_]^-^  307.1 (10) [M-H-C_6_H_10_O_5_-C_13_H_12_O_5_]^-^ | 307.1 (100) [M-H-C_6_H_10_O_5_-C_13_H_12_O_5_]^-^  247.0 (55) [M-H-C_6_H_10_O_5_- C_15_H_16_O_7_]^-^ |

Table S1 (Continued)

| **NO.** | **Peak** | ***t*_R_ (min)** | **Identification** | **Formula** | **[M+H]^+^/[M-H]^-^** | | **Error (ppm)** | **MS^2^ (%)** | **MS^3^ (%)** |
| --- | --- | --- | --- | --- | --- | --- | --- | --- | --- |
|  |  |  |  |  | **Pred. (*m/z*)** | **Meas. (*m/z*)** |  |  |  |
| 49 | L11 | 16.14 | Aloin A/B | C_21_H_22_O_9_ | 417.1172 | 417.1180 | -1.92 | 297.1 (100) [M-H-C_4_H_8_O_4_]^-^ | 268.1 (100) [M-H-C_4_H_8_O_4_-CHO]^-^  251.1 (56) [M-H-C_4_H_8_O_4_-CO-H_2_O]^-^  269.0 (56) [M-H-C_4_H_8_O_4_-CO]^-^  279.1 (33) [M-H-C_4_H_8_O_4_-H_2_O]^-^ |
| 50 | L12 | 16.50 | Aloeresin D or its isomer | C_29_H_32_O_11_ | 555.1849 | 555.1860 | -1.98 | 511.2 (100) [M-H-C_2_H_4_O]^-^  537.2 (50) [M-H-H_2_O]^-^ | 163.0 (100) [M-H-C_2_H_4_O-C_3_H_4_-C_15_H_16_O_7_]^-^  145.0 (64) [M-H-C_2_H_4_O-C_3_H_4_-C_15_H_16_O_7_-H_2_O]^-^  243.1 (22) [M-H-C_2_H_4_O-C_4_H_8_O_4_-C_9_H_8_O_2_]^-^ |
| 51 | L14 | 17.07 | Aloin A/B | C_21_H_22_O_9_ | 417.1177 | 417.1180 | -0.72 | 297.1 (100) [M-H-C_4_H_8_O_4_]^-^ | 268.1 (100) [M-H-C_4_H_8_O_4_-CHO]^-^  269.0 (64) [M-H-C_4_H_8_O_4_-CO]^-^  251.1 (60) [M-H-C_4_H_8_O_4_-CO-H_2_O]^-^ 279.0 (33) [M-H-C_4_H_8_O_4_-H_2_O]^-^ |

Table S1 (Continued)

| **NO.** | **Peak** | ***t*_R_ (min)** | **Identification** | **Formula** | **[M+H]^+^/[M-H]^-^** | | **Error (ppm)** | **MS^2^ (%)** | **MS^3^ (%)** |
| --- | --- | --- | --- | --- | --- | --- | --- | --- | --- |
|  |  |  |  |  | **Pred. (*m/z*)** | **Meas. (*m/z*)** |  |  |  |
| 52 | L15 | 17.18 | 7-*O*-Methylaloeresin A | C_29_H_30_O_11_ | 553.1697 | 553.1704 | -1.27 | 407.2 (100) [M-H-C_9_H_6_O_2_]^-^ | 243.1 (100) [M-H-C_9_H_6_O_2_-C_6_H_12_O_5_]^-^  365.2 (56) [M-H-C_9_H_6_O_2_-C_2_H_2_O]^-^ |
| 53 | L16 | 17.59 | Aloeresin D or its isomer | C_29_H_32_O_11_ | 555.1849 | 555.1860 | -1.98 | 511.2 (100) [M-H-C_2_H_4_O]^-^  537.2 (32) [M-H-H_2_O]^-^ | 163.0 (100) [M-H-C_2_H_4_O-C_3_H_4_-C_15_H_16_O_7_]^-^  145.0 (52) [M-H-C_2_H_4_O-C_3_H_4_-C_15_H_16_O_7_-H_2_O]^-^  243.1 (18) [M-H-C_2_H_4_O-C_4_H_8_O_4_-C_9_H_6_O-H_2_O]^-^ |
| 54 | L17 | 18.27 | Aloinoside A | C_27_H_32_O_13_ | 563.1757 | 563.1759 | -0.36 | 443.3 (100) [M-H-C_4_H_8_O_4_]^-^ | 425.1 (100) [M-H-C_4_H_8_O_4_-H_2_O]^-^  281.1 (40) [M-H-C_4_H_8_O_4_-C_6_H_10_O_5_]^-^  253.1 (29) [M-H-C_4_H_8_O_4_-C_6_H_10_O_5_-CO]^-^  251.1 (30) [M-H-C_4_H_8_O_4_-C_6_H_10_O_5_-CH_2_O]^-^ |
| 55 | L18 | 18.54 | Aloenin-2''-*p*-coumaroyl ester | C_28_H_28_O_12_ | 555.1493 | 555.1497 | -0.72 | 307.1 (100) [M-H-C_13_H_12_O_5_]^-^  247.1 (60) [M-H-C_15_H_16_O_7_]^-^ | 145.0 (100) [M-H-C_13_H_12_O_5_-C_6_H_10_O_5_]^-^  163.0 (37) [M-H-C_13_H_12_O_5_-C_9_H_7_O_3_]^-^ |

Table S1 (Continued)

| **NO.** | **Peak** | ***t*_R_ (min)** | **Identification** | **Formula** | **[M+H]^+^/[M-H]^-^** | | **Error (ppm)** | **MS^2^ (%)** | **MS^3^ (%)** |
| --- | --- | --- | --- | --- | --- | --- | --- | --- | --- |
|  |  |  |  |  | **Pred. (*m/z*)** | **Meas. (*m/z*)** |  |  |  |
| 56 | L19 | 18.72 | Aloeresin G | C_29_H_30_O_10_ | 537.1755 | 537.1755 | 0.00 | 163.0 (100) [M-H-C_5_H_6_-C_15_H_16_O_7_]^-^  269.2 (24) [M-H-C_9_H_8_O_2_-C_4_H_8_O_4_]^-^ | 119.0 (100) [M-H-C_5_H_6_-C_2_H_4_O]^-^ |
| 57 | L20 | 18.97 | 4-Hydroxymethyl-3-methoxyindole-2-*O*-glucoside or its isomer | C_23_H_24_O_10_ | 459.1289 | 459.1285 | 0.87 | 297.1 (100) [M-H-C_6_H_10_O_5_]^-^ | 268.1 (100) [M-H-C_6_H_10_O_5_-CHO]^-^  269.0 (63) [M-H-C_6_H_10_O_5_-CO]^-^  251.1 (58) [M-H-C_6_H_10_O_5_-CO-H_2_O]^-^  279.0 (32) [M-H-C_6_H_10_O_5_-H_2_O]^-^ |
| 58 | Z1 | 19.23 | Taurococholic acid or its isomer | C_26_H_45_NO_7_S | 514.2830 | 514.2832 | -0.39 | 514 (100) | 514 (100) |
| 59 | L21 | 19.65 | 4-Hydroxymethyl-3-methoxyindole-2-*O*-glucoside or its isomer | C_23_H_24_O_10_ | 459.1290 | 459.1285 | 1.09 | 297.1 (100) [M-H-C_6_H_10_O_5_]^-^ | 268.1 (100) [M-H-C_6_H_10_O_5_-CHO]^-^  269.0 (62) [M-H-C_6_H_10_O_5_-CO]^-^  251.1 (57) [M-H-C_6_H_10_O_5_-CO-H_2_O]^-^  279.0 (32) [M-H-C_6_H_10_O_5_-H_2_O]^-^ |
| 60 | Z2 | 20.07 | Taurochenodeoxycholic acid or its isomer | C_26_H_45_NO_6_S | 498.2887 | 498.2884 | 0.60 | 498.3 (100) | 498.3 (100) |

Table S1 (Continued)

| **NO.** | **Peak** | ***t*_R_ (min)** | **Identification** | **Formula** | **[M+H]^+^/[M-H]^-^** | | **Error (ppm)** | **MS^2^ (%)** | **MS^3^ (%)** |
| --- | --- | --- | --- | --- | --- | --- | --- | --- | --- |
|  |  |  |  |  | **Pred. (*m/z*)** | **Meas. (*m/z*)** |  |  |  |
| 61 | Z3 | 20.15 | Glyhyocholic acid | C_26_H_43_NO_6_ | 464.3004 | 464.3006 | -0.43 | 418.4 (100) [M-H-CO-H_2_O]^-^  400.4 (18) [M-H-CO-2H_2_O]]^-^  446.4 (18) [M-H-H_2_O]^-^  384.5 (14) [M-H-2H_2_O-CO_2_]^-^ | / |
| 62 | Z4 | 20.24 | Taurochenodeoxycholic acid or its isomer | C_26_H_45_NO_6_S | 498.2888 | 498.2884 | 0.80 | 498.3 (100) | 498.3 (100) |
| 63 | Z5 | 20.52 | Taurohyocholic acid or its isomer | C_26_H_45_NO_7_S | 514.2811 | 514.2832 | -4.08 | 514 (100) | 514 (100) |
| 64 | Z6 | 21.14 | Taurochenodeoxycholic acid or its isomer | C_26_H_45_NO_6_S | 498.2877 | 498.2884 | -1.40 | 498.3 (100) | 498.3 (100) |
| 65 | Z7 | 21.31 | Glychenodeoxycholic acid | C_26_H_43_NO_5_ | 448.3057 | 448.3057 | 0.00 | 386.3 (100) [M-H-H_2_O-CO_2_]^-^  404.4 (42) [M-H-CO_2_]^-^ | / |
| 66 | Z8 | 21.40 | Taurohyodeoxycholic acid or its isomer | C_26_H_45_NO_6_S | 498.286 8 | 498.2884 | -3.21 | 498.3 (100) | 498.3 (100) |
| 67 | Z9 | 21.42 | Glycocholic acid | C_26_H_43_NO_6_ | 464.3002 | 464.3006 | -0.86 | 420.3 (100) [M-H-CO_2_]^-^  389.3 (66) [M-H-C_2_H_5_NO_2_]^-^  384.5 (56) [M-H-2H_2_O-CO_2_]^-^  402.4 (35) [M-H-H_2_O-CO_2_]^-^  446.4 (23) [M-H-H_2_O]^-^ | 384.4 (100) [M-H-CO_2_-2H_2_O]^-^  402.4 (95) [M-H-H_2_O-CO_2_]^-^ |

Table S1 (Continued)

| **NO.** | **Peak** | ***t*_R_ (min)** | **Identification** | **Formula** | **[M+H]^+^/[M-H]^-^** | | **Error (ppm)** | **MS^2^ (%)** | **MS^3^ (%)** |
| --- | --- | --- | --- | --- | --- | --- | --- | --- | --- |
|  |  |  |  |  | **Pred. (*m/z*)** | **Meas. (*m/z*)** |  |  |  |
| 68 | Z10 | 22.30 | Glyhyodeoxycholic acid | C_26_H_43_NO_5_ | 448.3043 | 448.3057 | -3.12 | 386.3 (100) [M-H-H_2_O-CO_2_]^-^  404.4 (72) [M-H-CO_2_]^-^ | 368.4 (100) [M-H-2H_2_O-CO_2_]^-^ |
| 69 | Z11 | 22.59 | Nutriaglycocholic acid or its isomer | C_26_H_41_NO_5_ | 446.2898 | 446.2900 | -0.45 | 402.4 (100) [M-H-CO_2_]^-^ | / |
| 70 | Z12 | 22.89 | Nutriaglycocholic acid or its isomer | C_26_H_41_NO_5_ | 446.2898 | 446.2900 | -0.45 | 402.3 (100) [M-H-CO_2_]^-^  384.4 (20) [M-H-CO_2_-H_2_O]^-^ | 384.3 (100) [M-H-CO_2_-H_2_O]^-^ |
| 71 | Z13 | 23.08 | Nutriaglycocholic acid or its isomer | C_26_H_41_NO_5_ | 446.2903 | 446.2900 | 0.67 | 402.3 (100) [M-H-CO_2_]^-^ | / |
| 72 | Z14 | 23.41 | Cholic acid | C_24_H_40_O_5_ | 407.2789 | 407.2792 | -0.74 | 389.3 (100) [M-H-H_2_O]^-^ | 371.3 (100) [M-H-2H_2_O]^-^ |
| 73 | Z15 | 23.43 | Taurochenodeoxycholic acid or its isomer | C_26_H_45_NO_6_S | 498.2880 | 498.2884 | -0.80 | 498.3 (100) | 498.3 (100) |
| 74 | Z16 | 23.69 | Taurochenodeoxycholic acid or its isomer | C_26_H_45_NO_6_S | 498.2864 | 498.2884 | -4.01 | 498.3 (100) | 498.3 (100) |
| 75 | Z17 | 24.05 | Hyocholic acid | C_24_H_40_O_5_ | 407.2792 | 407.2792 | 0.00 | 343.2 (100) [M-H-CO-2H_2_O]^-^  345.2 (60) [M-H-CO_2_-H_2_O]^-^  353.4 (28) [M-H-3H_2_O]^-^  327.3 (19) [M-H-CO_2_-2H_2_O]^-^  389.4 (16) [M-H-H_2_O]^-^  371.3 (10) [M-H-2H_2_O]^-^ | 325.3 (100) [M-H-CO-3H_2_O]^-^ |

Table S1 (Continued)

| **NO.** | **Peak** | ***t*_R_ (min)** | **Identification** | **Formula** | **[M+H]^+^/[M-H]^-^** | | **Error (ppm)** | **MS^2^ (%)** | **MS^3^ (%)** |
| --- | --- | --- | --- | --- | --- | --- | --- | --- | --- |
|  |  |  |  |  | **Pred. (*m/z*)** | **Meas. (*m/z*)** |  |  |  |
| 76 | Z18 | 24.35 | Isoglychenodeoxycholic acid | C_26_H_43_NO_5_ | 448.3044 | 448.3057 | -2.90 | 386.4 (100) [M-H-H_2_O-CO_2_]^-^  404.4 (11) [M-H-CO_2_]^-^ | 330.3 (100) [M-H-H_2_O-CO_2_-C_4_H_8_]^-^  368.3 (14) [M-H-2H_2_O-CO_2_]- |
| 77 | Z19 | 26.05 | Taurolithocolic acid | C_26_H_45_NO_5_S | 482.2937 | 482.2935 | 0.41 | 482.3 (100) | 482.3 (100) |
| 78 | Z20 | 26.28 | Hyodeoxycholic acid | C_24_H_40_O_4_ | 391.2837 | 391.2842 | -1.28 | 373.3 (100) [M-H-H_2_O]^-^ | / |
| 79 | Z21 | 26.40 | Taurolithocolic acid | C_26_H_45_NO_5_S | 482.2936 | 482.2935 | 0.21 | 482.3 (100) | 482.3 (100) |
| 80 | C1 | 21.53 | Neoandrographolide | C2_6_H_40_O_8_ | 525.2672 | 525.2694  [M+HCOOH-H]^-^ | 4.19 | 479.3 (100) [M-H]^-^ | 317.2 (100) [M-H-C_6_H_10_O_5_]^-^  161.0 (64) [C_10_H_11_O_2_]^-^ |

Table S1 (Continued)

| **NO.** | **Peak** | ***t*_R_ (min)** | **Identification** | **Formula** | **[M+H]^+^/[M-H]^-^** | | | **Error (ppm)** | **MS^2^ (%)** | **MS^3^ (%)** |
| --- | --- | --- | --- | --- | --- | --- | --- | --- | --- | --- |
|  |  |  |  |  | **Pred. (*m/z*)** | **Meas. (*m/z*)** | |  |  |  |
| 81 | BC1 | 0.96 | Quinic acid | C_7_H_12_O_6_ | 191.0550 | | 191.0559 | -4.71 | 85.0 (100) [M-H-CO_2_-2H_2_O-C_2_H_2_]^-^  127.0 (99) [M-H-H_2_O-CH_2_O]^-^  173.0 (86) [M-H-H_2_O]^-^  93.0 (52) [M-H-CO_2_-3H_2_O]^-^  111.0 (43) [M-H-CO_2_-2H_2_O]^-^ | / |
| 82 | X2 | 2.56 | Unknown | C_7_H_14_O_5_ | 177.0757 | | 177.0760 | -1.69 | 101.0 (100)  118.7 (10) | 57.0 (100) |
| 83 | X3 | 3.69 | Vanillyl mandelic acid | C_9_H_10_O_5_ | 197.0444 | | 197.0453 | 4.57 | 179.0 (100) [M-H-H_2_O]^-^ | 134.9 (100) [M-H-H_2_O-CO_2_]^-^ |
| 84 | BDP4 | 3.80 | Protocatechuate | C_7_H_6_O_4_ | 153.0182 | | 153.0182 | 1.31 | 109.0 (100) [M-H-CO_2_]^-^ | 91.0 (100) [M-H-CO_2_-H_2_O]^-^  109.0 (78) [M-H-CO_2_]^-^ |
| 85 | P5 | 5.07 | Monocaffeoyltaric acid | C_13_H_12_O_9_ | 311.0397 | | 311.0395 | 0.64 | 149.0 (100) [M-H-C_9_H_6_O_3_]^-^ | 103.0 (100) [M-H-C_9_H_6_O_3_-CH_2_O_2_]^-^  87.0 (99) [M-H-C_9_H_6_O_3_-CO_2_-H_2_O]^-^  130.8 (46) [M-H-C_9_H_6_O_3_-H_2_O]^-^ |
| 86 | BC6 | 5.07 | 5-Caffeoylquinic acid | C_16_H_18_O_9_ | 353.0869 | | 353.0867 | 0.57 | 191.0 (100) [M-H-C_9_H_6_O_3_]^-^ | 127.0 (100) [M-H-C_9_H_6_O_3_-H_2_O-HCOOH]^-^  85.0 (94) [M-H-CO_2_-2H_2_O-C_2_H_2_]^-^  173.1 (66) [M-H-C_9_H_6_O_3_-H_2_O]^-^  93.0 (55) [M-H-C_9_H_6_O_3_-3H_2_O-CO_2_]^-^ 111.0 (30) [M-H-C_9_H_6_O_3_-2H_2_O-CO_2_]^-^ |

Table S1 (Continued)

| **NO.** | **Peak** | ***t*_R_ (min)** | **Identification** | **Formula** | **[M+H]^+^/[M-H]^-^** | | | **Error (ppm)** | **MS^2^ (%)** | **MS^3^ (%)** |
| --- | --- | --- | --- | --- | --- | --- | --- | --- | --- | --- |
|  |  |  |  |  | **Pred. (*m/z*)** | **Meas. (*m/z*)** | |  |  |  |
| 87 | P7 | 5.73 | Esculin | C_15_H_16_O_9_ | 339.0710 | | 339.0714 | -1.18 | 177.0 (100) [M-H-C_6_H_10_O_5_]^-^ | 133.0 (100) [M-H-C_6_H_10_O_5_-CO_2_]^-^ |
| 88 | X8 | 5.89 | Viridifloric acid or its isomer | C_7_H_14_O_4_ | 161.0808 | | 161.0810 | -1.24 | 117.0 (100) [M-H-CO_2_]^-^  99.0 (29) [M-H-H_2_O-CO_2_]^-^  71.0 (12) [M-H-H_2_O-CO_2_-CO]^-^ | 71.0 (100) [M-H-H_2_O-CO_2_-CO]^-^ |
| 89 | B9 | 6.12 | Gibboside | C_16_H_26_O_9_ | 407.1553 | | 407.1547 | 1.47 | 361.1 (100) [M-H]^-^ | 199.1 (100) [M-H-C_6_H_10_O_5_]^-^  161.0 (67) [M-H-C_6_H_10_O_5_-H_2_O]^-^ |
| 90 | X10 | 6.27 | Viridifloric acid or its isomer | C_7_H_14_O_4_ | 161.0808 | | 161.0810 | -1.24 | 117.0 (100) [M-H-CO_2_]^-^  99.0 (29) [M-H-H_2_O-CO_2_]^-^  71.0 (10) [M-H-H_2_O-CO_2_-CO]^-^ | 71.0 (100) [M-H-H_2_O-CO_2_-CO]^-^ |
| 91 | P11 | 6.51 | *p*-Coumaric acid | C_9_H_8_O_3_ | 163.0389 | | 163.0393 | -2.45 | 119.0 (100) [M-H-CO_2_]^-^ | 91.0 (100) [M-H-CO_2_-H_2_O]^-^ |
| 92 | BCP12 | 6.69 | Chlorogenic acid | C_16_H_18_O_9_ | 353.0863 | | 353.0867 | -1.13 | 191.0 (100) [M-H-C_9_H_6_O_3_]^-^ | 127.0 (100) [M-H-C_9_H_6_O_3_-H_2_O-2H-CO_2_]^-^  85.0 (95) [M-H-CO_2_-2H_2_O-C_2_H_2_]^-^  173.0 (68) [M-H-C_9_H_6_O_3_-H_2_O]^-^  93.0 (49) [M-H-C_9_H_6_O_3_-3H_2_O-CO_2_]^-^  111.0 (45) [M-H-C_9_H_6_O_3_-2H_2_O-CO_2_]^-^ |
| 93 | DP13 | 6.88 | Esculetin | C_9_H_6_O_4_ | 177.0182 | | 177.0182 | 0.00 | 133.0 (100) [M-H-CO_2_]^-^ | 105.0 (100) [M-H-CO_2_-CO]^-^ |
| 94 | XD14 | 7.14 | Caffeic acid | C_9_H_8_O_4_ | 179.0338 | | 179.0346 | -4.47 | 135.0 (100) [M-H-CO_2_]^-^  107.0 (55) [M-H-C_3_H_4_O_2_]^-^ | 135.0 (100) [M-H-CO_2_]^-^  107.0 (98) [M-H-C_3_H_4_O_2_]^-^ |

Table S1 (Continued)

| **NO.** | **Peak** | ***t*_R_ (min)** | **Identification** | **Formula** | **[M+H]^+^/[M-H]^-^** | | | **Error (ppm)** | **MS^2^ (%)** | **MS^3^ (%)** |
| --- | --- | --- | --- | --- | --- | --- | --- | --- | --- | --- |
|  |  |  |  |  | **Pred. (*m/z*)** | **Meas. (*m/z*)** | |  |  |  |
| 95 | CP15 | 7.21 | 4-Caffeoylquinic acid | C_16_H_18_O_9_ | 353.0863 | | 353.0867 | -1.13 | 173.0 (100) [M-H-C_9_H_6_O_3_-H_2_O]^-^  179.0 (54) [M-H-C_7_H_10_O_5_]^-^  191.0 (16) [M-H-C_9_H_6_O_3_]^-^ | 93.0 (100) [M-H-C_9_H_6_O_3_-3H_2_O-CO_2_]^-^  111.0 (60) [M-H-C_9_H_6_O_3_-2H_2_O-CO_2_]^-^  155.0 (16) [M-H-C_9_H_6_O_3_-2H_2_O]^-^ |
| 96 | L16 | 8.84 | Aloenin-10-*O*-*β*-D-glucopyranoside | C_25_H_32_O_15_ | 617.1712 | | 617.1712 | 0.00 | 409.0 (100) [M-H-C_6_H_10_O_5_]^-^  571.0 (9) [M-H]^-^ | 247.0 (100) [M-H-2C_6_H_10_O_5_]^-^ |
| 97 | B17 | 9.06 | Sweroside | C_16_H_22_O_9_ | 403.1234 | | 403.1235 | -0.25 | 357.2 (100) [M-H]^-^  195.1 (54) [M-H-C_6_H_10_O_5_]^-^  179.0 (42) [M-H-C_6_H_10_O_5_-H_2_O]^-^  125.0 (22) | 195.2 (100) [M-H-C_6_H_10_O_5_]^-^  125.0 (92) [M-H-C_6_H_10_O_5_-H_2_O- CO_2_]^-^ |
| 98 | C18 | 9.15 | Isovitexin -8-*C*-glucoside | C_27_H_30_O_15_ | 593.1499 | | 593.1500 | -0.17 | 473.2 (100) [M-H-C_4_H_8_O_4_]^-^  353.1 (48) [M-H-2C_4_H_8_O_4_]^-^  575.3 (10) [M-H-H_2_O]^-^ | 353.2 (100) [M-H-2C_4_H_8_O_4_]^-^ |
| 99 | B19 | 10.05 | 5,7,3’-Trihydroxy-4’-*O*-rhamnoylflavon-3-*O*-rutinoside | C_33_H_40_O_20_ | 755.2028 | | 755.2029 | -0.13 | 609.2 (100) [M-H-C_6_H_10_O_4_]^-^ | 301.1 (100) [M-H-C_12_H_20_O_9_]^-^ |

Table S1 (Continued)

| **NO.** | **Peak** | ***t*_R_ (min)** | **Identification** | **Formula** | **[M+H]^+^/[M-H]^-^** | | | **Error (ppm)** | **MS^2^ (%)** | **MS^3^ (%)** |
| --- | --- | --- | --- | --- | --- | --- | --- | --- | --- | --- |
|  |  |  |  |  | **Pred. (*m/z*)** | **Meas. (*m/z*)** | |  |  |  |
| 100 | B20 | 10.39 | 6*β*-Hydroxygeniposid | C_17_H_24_O_11_ | 403.1234 | | 403.1235 | -0.25 | 371.1 (100) [M-H-CH_3_-OH]^-^  223.0 (36) [M-H-H_2_O-C_6_H_10_O_5_]^-^  179.0 (18) [M-H-CH_3_-OH-C_6_H_10_O_5_-CO_2_]^-^ | 121.0 (100) [M-H-CH_3_-OH-C_6_H_10_O_5_-2CO_2_]^-^  165.0 (65) [M-H-CH_3_-OH-C_6_H_10_O_5_-CO_2_]^-^  209.0 (16) [M-H-CH_3_-OH-C_6_H_10_O_5_]^-^ |
| 101 | L21 | 10.79 | 5-(2'-Oxo-4'-hydroxypentyl)-2-(β-glucopyranosyl-oxy-methyl)chromone | C_21_H_26_O_10_ | 437.1442 | | 437.1442 | 0.00 | 391.1 (100) [M-H-H_2_O-CO]^-^ | 269.0 (100) [M-H-H_2_O-CO-2H-C_4_H_8_O_4_]^-^ |
| 102 | L22 | 10.88 | Isoaloenin | C_19_H_22_O_10_ | 409.1129 | | 409.1133 | -0.98 | 247.0 (100) [M-H-C_6_H_10_O_5_]^-^ | 203.0 (100) [M-H-C_6_H_10_O_5_-CO_2_]^-^  171.0 (27) [M-H-C_6_H_10_O_5_-CO_2_-CH_4_O]^-^  215.0 (25) [M-H-C_6_H_10_O_5_-CH_4_O]^-^ |
| 103 | B23 | 10.91 | Robinin | C_33_H_40_O_19_ | 739.2075 | | 739.2080 | -0.68 | 593.3 (100) [M-H-C_6_H_10_O_4_]^-^ | 285.1 (100) [M-H-C_12_H_20_O_9_]^-^ |
| 104 | C24 | 11.17 | Violanthin | C_27_H_30_O_14_ | 577.1553 | | 577.1552 | 0.17 | 269.0 (100) [M-H-C_12_H_20_O_9_]^-^ | 269.1 (100) [M-H-C_12_H_20_O_9_]^-^  151.0(100) [M-H-C_12_H_20_O_9_-C_7_H_5_O]^-^ |
| 105 | BDP25 | 11.60 | Rutin | C_27_H_30_O_16_ | 609.1448 | | 609.1450 | -0.33 | 301.1 (100) [M-H-C_12_H_20_O_9_]^-^  300.0 (29) [M-H-C_12_H_21_O_9_]^-^ | 151.0 (100) [M-H-C_12_H_20_O_9_-C_8_H_8_O_6_]^-^ |

Table S1 (Continued)

| **NO.** | **Peak** | ***t*_R_ (min)** | **Identification** | **Formula** | **[M+H]^+^/[M-H]^-^** | | | **Error (ppm)** | **MS^2^ (%)** | **MS^3^ (%)** |
| --- | --- | --- | --- | --- | --- | --- | --- | --- | --- | --- |
|  |  |  |  |  | **Pred. (*m/z*)** | **Meas. (*m/z*)** | |  |  |  |
| 106 | L26 | 12.09 | Aloenin | C_19_H_22_O_10_ | 409.1129 | | 409.1132 | -0.98 | 247.0 (100) [M-H-C_6_H_10_O_5_]^-^ | 203.1 (100) [M-H-C_6_H_10_O_5_-CO_2_]^-^  171.0 (28) [M-H-C_6_H_10_O_5_-CO_2_-CH_4_O]^-^  215.0 (23) [M-H-C_6_H_10_O_5_-CH_4_O]^-^ |
| 107 | DP27 | 12.20 | Kaempferol-7-*O*-glucopyranoside | C_21_H_20_O_11_ | 447.0922 | | 447.0928 | -1.34 | 285.1 (100) [M-H-C_6_H_10_O_5_]^-^ | 241.0 (100) [M-H-C_6_H_10_O_5_]^-^  151.0 (25) [M-H-C_6_H_10_O_5_-C_8_H_6_O_2_]^-^  257.1 (25) [M-H-C_6_H_10_O_5_-CO]^-^ |
| 108 | C28 | 13.30 | 3,4-Dicaffeoylquinic acid | C_25_H_24_O_12_ | 515.1188 | | 515.1184 | 0.78 | 353.1 (100) [M-H-C_9_H_6_O_3_]^-^  173.0 (14) [M-H- 2C_9_H_6_O_3_-H_2_O]^-^  335.1 (13) [M-H-C_9_H_6_O_3_-H_2_O]^-^ | 173.0 (100)  [M-H-2C_9_H_6_O_3_-H_2_O]^-^  179.1 (66) [M-H-C_9_H_6_O_3_-C_7_H_10_O_5_]^-^  191.0 (49) [M-H-2C_9_H_6_O_3_]^-^ |
| 109 | C29 | 13.55 | 3,5-Dicaffeoylquinic acid | C_25_H_24_O_12_ | 515.1188 | | 515.1184 | 0.78 | 353.1 (100) [M-H-C_9_H_6_O_3_]^-^ | 191.0 (100) [M-H-2C_9_H_6_O_3_]^-^  179.1 (58) [M-H-C_9_H_6_O_3_-C_7_H_10_O_5_]^-^  173.0 (43)  [M-H- 2C_9_H_6_O_3_-H_2_O]^-^ |
| 110 | L30 | 13.57 | 8-(*α*-*L*-Rhamnopyranosyloxy)-3-(*β*-dxylopyranosyloxymethyl)naphthalen-ol | C_24_H_30_O_12_ | 509.1653 | | 509.1653 | 0.00 | 363.1 (100) [M-H-C_6_H_10_O_4_]^-^ | 213.0 (100) [M-H-C_6_H_10_O_4_-C_5_H_10_O_5_]^-^ |

Table S1 (Continued)

| **NO.** | **Peak** | ***t*_R_ (min)** | **Identification** | **Formula** | **[M+H]^+^/[M-H]^-^** | | | **Error (ppm)** | **MS^2^ (%)** | **MS^3^ (%)** |
| --- | --- | --- | --- | --- | --- | --- | --- | --- | --- | --- |
|  |  |  |  |  | **Pred. (*m/z*)** | **Meas. (*m/z*)** | |  |  |  |
| 111 | X31 | 13.68 | Salvianolic acid B | C_36_H_30_O_16_ | 717.1450 | | 717.1449 | -0.14 | 519.1 (100) [M-H-C_9_H_9_O_6_]^-^  475.2 (50) [M-H-C_9_H_9_O_6_-CO_2_]^-^ | / |
| 112 | C32 | 14.60 | Apigenin-7-*O*-glucuronide | C_21_H_18_O_11_ | 445.0765 | | 445.0765 | 0.00 | 269.0 (100) [M-H-C_6_H_8_O_6_]^-^ | 225.0 (100) [M-H-C_6_H_8_O_6_-CO_2_]^-^  151.0 (25) [M-H-C_6_H_8_O_6_-C_6_H_8_O]^-^ |
| 113 | C33 | 15.22 | 4,5-Dicaffeoylquinic acid | C_25_H_24_O_12_ | 515.1186 | | 515.1184 | 0.39 | 353.2 (100) [M-H-C_9_H_6_O_3_]^-^ | 173.0 (100)  [M-H- 2C_9_H_6_O_3_-H_2_O]^-^  179.0 (62) [M-H-C_9_H_6_O_3_-C_7_H_10_O_5_]^-^  190.9 (29) [M-H-2C_9_H_6_O_3_]^-^ |
| 114 | C34 | 15.30 | 1,3,15,19-Tetrahydroxy-8(17),13-ent-labdadien-16-oic acid | C_20_H_32_O_6_ | 367.2118 | | 367.2115 | 0.82 | 349.3 (100) [M-H-H_2_O]^-^  305.3 (73) [M-H-H_2_O-CO_2_]^-^  287.2 (10) [M-H-2H_2_O-CO_2_]^-^ | 305.3 (100) [M-H-H_2_O-CO_2_]^-^ |
| 115 | B35 | 15.54 | Quercetin-7,4'-*di*-*O*-rhamnose-3-*O*-rutinoside | C_39_H_50_O_24_ | 901.2600 | | 901.2608 | -0.89 | 755.3 (100) [M-H-C_6_H_10_O_4_]^-^ | 609.3 (100) [M-H-2C_6_H_10_O_4_]^-^  301.0 (23) [M-H-2C_6_H_10_O_4_-C_12_H_20_O_9_]^-^ |
| 116 | C36 | 16.03 | 5,4'-Dihydroxy-7-methoxyflavone-6/8-*O*-pyranoglucoside | C_22_H_22_O_11_ | 461.1084 | | 461.1078 | 1.30 | 299.0 (100) [M-H-C_6_H_10_O_5_]^-^ | 284.0 (100) [M-H-C_6_H_10_O_5_-CH_3_]^-^ |
| 117 | C37 | 18.15 | Andrographolide | C_20_H_30_O_5_ | 395.2070 | | 395.2064  [M+HCOOH-H]^-^ | 1.52 | 331.2 (100) [M-H-H_2_O]^-^  349.0 (33) [M-H]^-^ | 303.2（100）[M-H-H_2_O-CO]^-^  288.2（71）[M-H-H_2_O-CO-CH_3_]^-^ |

Table S1 (Continued)

| **NO.** | **Peak** | ***t*_R_ (min)** | **Identification** | **Formula** | **[M+H]^+^/[M-H]^-^** | | | **Error (ppm)** | **MS^2^ (%)** | **MS^3^ (%)** |
| --- | --- | --- | --- | --- | --- | --- | --- | --- | --- | --- |
|  |  |  |  |  | **Pred. (*m/z*)** | **Meas. (*m/z*)** | |  |  |  |
| 118 | C38 | 18.47 | 12-Hydroxyandrographolide | C_20_H_30_O_6_ | 365.1962 | | 365.1959 | 0.82 | 321.3 (100) [M-H-CO_2_]^-^ | 277.3 (100) [M-H-CO_2_-HCOCH_3_]^-^ |
| 119 | C39 | 18.68 | Andrographic acid | C_20_H_28_O_6_ | 363.1808 | | 363.1802 | 1.65 | 319.4 (100) [M-H-CO_2_]^-^ | 227.2 (100) [M-H-2CO_2_-H_2_O-2CH_3_]^-^  245.3 (13) [M-H-2CO_2_-2CH_3_]^-^ |
| 120 | C40 | 19.25 | 14-Deoxyandrographoside | C_26_H_40_O_9_ | 541.2650 | | 541.2643  [M+HCOOH-H]^-^ | 1.29 | 495.2592 (100) [M-H]^-^ | 333.2 (100) [M-H-C_6_H_10_O_5_]^-^  161.0 (10) [C_10_H_11_O_2_]^-^ |
| 121 | L41 | 19.57 | Aloe emodin | C_15_H_10_O_5_ | 269.0449 | | 269.0444 | 1.86 | 269.0 (100) [M-H]^-^  225.0 (58) [M-H-CO-O]^-^  241.0 (29) [M-H-CO]^-^ | / |
| 122 | C42 | 19.62 | 14-Deoxy-11,12-didehydroandrographoside | C_26_H_38_O_9_ | 539.2491 | | 539.2486  [M+HCOOH-H]^-^ | 0.93 | 493.3 (100) [M-H]^-^ | 331.0 (100) [M-H-C_6_H_10_O_5_]^-^ |
| 123 | C43 | 19.91 | 14-Deoxyandrographoside | C_26_H_40_O_9_ | 541.2651 | | 541.2643  [M+HCOOH-H]^-^ | 1.48 | 495.2592 (100) [M-H]^-^  333.3 (24) [M-H-C_6_H_10_O_5_]^-^ | 333.2 (100) [M-H-C_6_H_10_O_5_]^-^  161.0 (10) [C_10_H_11_O_2_]^-^ |

**K**: the ingredients come from Sophorae Flavescentis Radix (SFR); **L**: the ingredients come from Aloe; **Z**: the ingredients come from Suis Fellis Pulvis (SFP); **C**: the ingredients come from Suis Fellis Pulvis (SFP); **B:** the ingredients come from Herba Patriniae (HP); **D:** the ingredients come from Violsse Herba (VH); X: the ingredients come from Arnebiae Radix (AR); **P:** the ingredients come from Taraxaci Herba (TH).

**Table S2 The ingredients absorbed into blood of KFXYS**

| **NO.** | **Identification** | **Formula** | ***t*_R_ (min)** | **[M+H]^+^/[M-H]^-^** | | **Error (ppm)** | **Response** | **MS/MS** | **Type** |
| --- | --- | --- | --- | --- | --- | --- | --- | --- | --- |
|  |  |  |  | **Meas. (*m/z*)** | **Pred. (*m/z*)** |  |  |  |  |
| **Alkaloids** | | | | | | | | | |
| 1 | N-Methylcytisine | C_12_H_16_N_2_O | 1.43 | 205.1331 | 205.1341 | -4.87 | 3023 | 118.0649 [M+H-C_3_H_9_N-CO]^+^ | P |
| 2 | 5-Hydroxysparteine | C_15_H_24_N_2_O_2_ | 1.87 | 265.1902 | 265.1916 | -5.28 | 1580 | 164.1094 [M+H-2H-C_6_H_13_N]^+^  146 [M+H-2H-H_2_O-C_6_H_13_N]^+^ | P |
| 3 | Hydroxymatrine | C_15_H_24_N_2_O_2_ | 2.21 | 265.1901 | 265.1916 | -5.66 | 3774 | 247.1674 [M+H-H_2_O]^+^  150.1226 [M+H-C_5_H_7_NO]^+^  148.1125 [M+H-C_5_H_9_NO]^+^ | P |
| 4 | Matrine-2H | C_15_H_22_N_2_O | 2.40 | 247.1798 | 247.1810 | -4.85 | 1913 | 148.1125 [M+H-2H-C_5_H_7_NO]^+^  150.1280 [M+H-C_5_H_7_NO]^+^ | M |
| 5 | Matrine-2H | C_15_H_22_N_2_O | 2.65 | 247.1798 | 247.1810 | -4.85 | 3624 | 245.1671 [M+H-2H]^+^  148.1125 [M+H-2H-C_5_H_7_NO]^+^  150.1280 [M+H-C_5_H_7_NO]^+^ | M |
| 6 | Matrine | C_15_H_24_N_2_O | 2.84 | 249.1957 | 249.1967 | -4.01 | 93503 | 247.1812[M+H-2H]^+^  148.1125 [M+H-2H-C_5_H_7_NO]^+^  150.1299 [M+H-C_5_H_7_NO]^+^  120.0802 [M+H-2H-C_5_H_7_NO-CO]^+^ | P |

Table S2 (Continued)

| **NO.** | **Identification** | **Formula** | ***t*_R_ (min)** | **[M+H]^+^/[M-H]^-^** | | **Error (ppm)** | **Response** | **MS/MS** | **Type** |
| --- | --- | --- | --- | --- | --- | --- | --- | --- | --- |
|  |  |  |  | **Meas. (*m/z*)** | **Pred. (*m/z*)** |  |  |  |  |
| 7 | Sophocarpine | C_15_H_22_N_2_O | 3.07 | 247.1794 | 247.1810 | -6.47 | 14091 | 245.1671 [M+H-2H]^+^  179.1531 [M+H-C_4_H_4_O]^+^  136.1127 [M+H-2H-C_6_H_7_NO]^+^  148.1125 [M+H-2H-C_5_H_7_NO]^+^  150.1299 [M+H-C_5_H_7_NO]^+^ | P |
| 8 | Isomatrine | C_15_H_24_N_2_O | 3.21 | 249.1955 | 249.1967 | -4.82 | 3697 | 247.1812[M+H-2H]^+^  120.0802 [M+H-2H-C_5_H_7_NO-CO]^+^ | P |
| 9 | 13,14-Dehydrolupanine | C_15_H_22_N_2_O | 3.47 | 247.1796 | 247.1810 | -5.66 | 782 | 112.0773 [M+H-C_9_H_13_N]^+^ | P |
| 10 | Cytisine+O-2H | C_11_H_12_N_2_O_2_ | 4.19 | 205.0963 | 205.0977 | -6.83 | 44114 | 146.0633 [M+H-C_2_H_5_NO]^+^  118.0649 [M+H-C_2_H_5_NO-CO]^+^  130.0637 [M+H-C_2_H_5_NO-O]^+^  128.0500 [M+H-C_2_H_5_NO-H_2_O]^+^ | M |
| 11 | Matrine-2H+2O | C_15_H_22_N_2_O_3_ | 4.19 | 279.1695 | 279.1709 | -5.01 | 435 | 261.1642 [M+H-H_2_O]^+^  243.1477 [M+H-2H_2_O]^+^  148.1125 [M+H-2H_2_O]^+^ | M |
| 12 | 5,6-Dehydrolupanine | C_15_H_22_N_2_O | 4.40 | 247.1794 | 247.1810 | -6.47 | 2044 | 148.1125[M+H-C_6_H_13_N]^+^  120.0802 (65) [M+H-C_6_H_13_N-CO]^+^ | P |
| 13 | Cytisine+O-2H | C_11_H_12_N_2_O_2_ | 6.83 | 205.0963 | 205.0977 | -6.83 | 625 | 146.0633 [M+H-C_2_H_5_NO]^+^  118.0649 [M+H-C_2_H_5_NO-CO]^+^ | M |

Table S2 (Continued)

| **NO.** | **Identification** | **Formula** | ***t*_R_ (min)** | **[M+H]^+^/[M-H]^-^** | | **Error (ppm)** | **Response** | **MS/MS** | **Type** |
| --- | --- | --- | --- | --- | --- | --- | --- | --- | --- |
|  |  |  |  | **Meas. (*m/z*)** | **Pred. (*m/z*)** |  |  |  |  |
| 14 | 5-Hydroxysparteine+O-2H | C_15_H_22_N_2_O_3_ | 6.84 | 279.1689 | 279.1709 | -7.16 | 409 | 146.0633 [M+H-C_6_H_11_N]^+^  118.0649 [M+H-C_6_H_11_N-CO]^+^ | M |
| 15 | Matrine-2H+O | C_15_H_22_N_2_O_2_ | 7.89 | 263.1744 | 263.1760 | -6.08 | 3207 | 261.1642 [M+H-2H]^+^  245.1671 [M+H-H_2_O]^+^ | M |
| 16 | Matrine-2H+O | C_15_H_22_N_2_O_2_ | 9.28 | 263.1747 | 263.1760 | -4.94 | 14427 | 245.1671 [M+H-H_2_O]^+^  148.1125 [M+H-C_5_H_5_NO-2H-H_2_O]^+^  150.1280 [M+H-C_5_H_7_NO-H_2_O]^+^ | M |
| **Flavonoids** | | | | | | | | | |
| 17 | Kaempferol+2H+C_6_H_8_O_6_ | C_21_H_20_O_12_ | 5.09 | 463.0874 | 463.0877 | -0.65 | 2422 | 287.0558 [M-H-C_6_H_8_O_6_]^-^  259.0600 [M-H-C_6_H_8_O_6_-CO]^-^  135.0457 [M-H-C_6_H_8_O_6_-C_7_H_4_O]^-^ | M |
| 18 | Kaempferol+2H+C_6_H_8_O_6_ | C_21_H_20_O_12_ | 5.54 | 463.0876 | 463.0877 | -0.22 | 796 | 287.0570 [M-H-C_6_H_8_O_6_]^-^  259.0600 [M-H-C_6_H_8_O_6_-CO]^-^  135.0457 [M-H-C_6_H_8_O_6_-C_7_H_4_O]^-^ | M |
| 19 | 5,7,4'-Trihydroxyisoflavone+O+2H+C_6_H_8_O_6_ | C_21_H_20_O_12_ | 6.33 | 465.1014 | 465.1033 | -4.09 | 716 | 289.0729 [M+H-C_6_H_8_O_6_]^+^  271.0639 [M+H-C_6_H_8_O_6_-H_2_O]^+^ | M |

Table S2 (Continued)

| **NO.** | **Identification** | **Formula** | ***t*_R_ (min)** | **[M+H]^+^/[M-H]^-^** | | **Error (ppm)** | **Response** | **MS/MS** | **Type** |
| --- | --- | --- | --- | --- | --- | --- | --- | --- | --- |
|  |  |  |  | **Meas. (*m/z*)** | **Pred. (*m/z*)** |  |  |  |  |
| 20 | Kaempferol+2H+C_6_H_8_O_6_+CH_2_ | C_22_H_22_O_12_ | 7.03 | 477.1034 | 477.1033 | 0.21 | 1015 | 301.0712 [M-H-C_6_H_8_O_6_]^-^  269.0421 [M-H-C_6_H_8_O_6_-CH_2_-H_2_O]^-^  165.0161 [M-H-C_6_H_8_O_6_-C_8_H_8_O_2_]^-^ | M |
| 21 | Formononetim-CH_2_+C_6_H_8_O_6_ | C_21_H_18_O_10_ | 8.26 | 431.0985 | 431.0978 | 1.62 | 4549 | 255.0616 [M+H-C_6_H_8_O_6_]^+^  237.0546 [M+H-C_6_H_8_O_6_-H_2_O]^+^ | M |
| 22 | Kaempferol+C_6_H_8_O_6_ | C_21_H_18_O_12_ | 8.74 | 461.0741 | 461.0720 | 4.55 | 835 | 285.0419 [M-H-C_6_H_8_O_6_]^-^  241.0503 [M-H-C_6_H_10_O_6_-O-CO]^-^ | M |
| 23 | 7,4'-Dihydroxy-3'-methoxyisoflavone+C_6_H_8_O_6_ | C_22_H_20_O_11_ | 8.75 | 461.1059 | 461.1084 | -5.42 | 1183 | 285.0744 [M+H-C_6_H_8_O_6_]^+^ | M |
| 24 | Apigenin-O+C_6_H_8_O_6_ | C_21_H_18_O_10_ | 8.84 | 429.0811 | 429.0822 | -2.56 | 1456 | 253.0501 [M-H-C_6_H_8_O_6_]^-^ | M |
| 25 | Formononetin-CH_2_+2H | C_15_H_12_O_4_ | 10.19 | 257.0803 | 257.0814 | -4.28 | 2893 | 165.0597 [M+H-C_6_H_4_O]^+^ | M |
| 26 | 5,7,4'-Trihydroxyisoflavone+C_6_H_8_O_6_ | C_21_H_18_O_11_ | 10.57 | 447.0909 | 447.0927 | -4.03 | 4141 | 271.0597 [M+H-C_6_H_8_O_6_]^+^ | M |
| 27 | Apigenin-O+4H+C_6_H_8_O_6_ | C_21_H_22_O_10_ | 11.06 | 433.1132 | 433.1135 | -0.69 | 6686 | 257.0829 [M-H-C_6_H_8_O_6_]^-^  109.0277 [M-H-C_6_H_8_O_6_-C_9_H_8_O_2_]^-^  121.0680 [M-H-C_6_H_8_O_6_-C_7_H_4_O_3_]^-^ | M |

Table S2 (Continued)

| **NO.** | **Identification** | **Formula** | ***t*_R_ (min)** | **[M+H]^+^/[M-H]^-^** | | **Error (ppm)** | **Response** | **MS/MS** | **Type** |
| --- | --- | --- | --- | --- | --- | --- | --- | --- | --- |
|  |  |  |  | **Meas. (*m/z*)** | **Pred. (*m/z*)** |  |  |  |  |
| 28 | Apigenin+C_6_H_8_O_6_ | C_21_H_18_O_11_ | 11.19 | 445.0771 | 445.0771 | 0.00 | 3185 | 269.045 [M-H-C_6_H_8_O_6_]^-^,  225.0574 [M-H-C_6_H_8_O_6_-CO_2_]^-^ | M |
| 29 | Formononetin-CH_2_+2H+C_6_H_8_O_6_+SO_3_ | C_21_H_20_O_13_S | 12.41 | 513.0693 | 513.0703 | -1.95 | 1513 | 239.0732 [M+H-C_6_H_8_O_6_-SO_3_-H_2_O]^+^  165.0597[M+H-C_6_H_8_O_6_-SO_3_-H_2_O-C_6_H_4_O]^+^ | M |
| 30 | 5,7,4'-Trihydroxyisoflavone+2H+C_6_H_8_O_6_ | C_21_H_20_O_11_ | 12.58 | 449.1056 | 449.1084 | -6.23 | 651 | 273.075 [M+H-C_6_H_8_O_6_]^+^ | M |
| 31 | Formononetin-CH_2_+4H+C_6_H_8_O_6_ | C_21_H_22_O_10_ | 16.36 | 435.1268 | 435.1291 | -5.29 | 421 | 259.0956 [M+H-C_6_H_8_O_6_]^+^  149.0597[M+H-C_6_H_8_O_6_-C_5_H_6_O_2_]^+^ | M |
| 32 | 7,4'-Dihydroxy-3'-methoxyisoflavone+C_6_H_8_O_6_ | C_22_H_20_O_11_ | 17.13 | 461.1052 | 461.1084 | -6.94 | 719 | 285.0747 [M+H-C_6_H_8_O_6_]^+^ | M |
| 33 | 4'-methoxyisoflavone-7-O-glucuronide | C_22_H_20_O_10_ | 25.49 | 445.1144 | 445.1135 | 2.02 | 1705 | 285.0750 [M+H-C_6_H_7_O_5_]^+^  268.0730 [M+H-C_6_H_7_O_5_-OH]^+^ | M |

Table S2 (Continued)

| **NO.** | **Identification** | **Formula** | ***t*_R_ (min)** | **[M+H]^+^/[M-H]^-^** | | **Error (ppm)** | **Response** | **MS/MS** | **Type** |
| --- | --- | --- | --- | --- | --- | --- | --- | --- | --- |
|  |  |  |  | **Meas. (*m/z*)** | **Pred. (*m/z*)** |  |  |  |  |
| 34 | 7,4'-Dihydroxy-3'-methoxyisoflavone+2H | C_16_H_14_O_5_ | 19.39 | 287.0908 | 287.0919 | -3.83 | 1470 | 269.0798 [M+H-H_2_O]^+^ | M |
| **Organic acids** | | | | | | | | | |
| 35 | Caffeic acid acid+SO_3_ | C_9_H_8_O_7_S | 2.71 | 258.9912 | 258.9912 | 0.00 | 507 | 179.0331 [M-H-SO_3_]^-^  135.0205 [M-H-SO_3_-CO_2_]^-^ | M |
| 36 | Caffeic acid+H2+SO_3_ | C_9_H_10_O_7_S | 3.51 | 261.0066 | 261.0069 | -1.15 | 1355 | 181.0504 [M-H-SO_3_]^-^  163.0408 [M-H-H_2_O]^-^  137.0590 [M-H-CO_2_]^-^ | M |
| 37 | Caffeic acid+H2+SO_3_ | C_9_H_10_O_7_S | 4.13 | 261.0069 | 261.0069 | 0.00 | 1470 | 181.0504 [M-H-SO_3_]^-^  163.0408 [M-H-H_2_O]^-^  137.0590 [M-H-CO_2_]^-^ | M |
| 38 | Caffeic acid+2H | C_9_H_10_O_4_ | 4.33 | 181.0504 | 181.0501 | 1.66 | 3153 | 163.0408 [M-H-H_2_O]^-^  137.0590 [M-H-CO_2_]^-^  109.0277 [M-H-C_3_H_4_O_2_]^-^ | M |
| 39 | Caffeic acid+C_6_H_8_O_6_ | C_15_H_16_O_10_ | 4.35 | 355.0665 | 355.0665 | 0.00 | 1024 | 175.0613 [M-H-C_6_H_8_O_6_-H_2_O]^-^ | M |
| 40 | Caffeic acid+2H | C_9_H_10_O_4_ | 4.59 | 181.0504 | 181.0501 | 1.66 | 13437 | 163.0408 [M-H-H_2_O]^-^  137.0639 [M-H-CO_2_]^-^  109.0277 [M-H-C_3_H_4_O_2_]^-^ | M |

Table S2 (Continued)

| **NO.** | **Identification** | **Formula** | ***t*_R_ (min)** | **[M+H]^+^/[M-H]^-^** | | **Error (ppm)** | **Response** | **MS/MS** | **Type** |
| --- | --- | --- | --- | --- | --- | --- | --- | --- | --- |
|  |  |  |  | **Meas. (*m/z*)** | **Pred. (*m/z*)** |  |  |  |  |
| 41 | Ferulic acid+SO_3_ | C_10_H_10_O_7_S | 5.34 | 273.0056 | 273.0069 | -4.76 | 1313 | 193.0511 [M-H-SO_3_]^-^,  134.0372 [M-H-SO_3_-CH_3_]^-^ | M |
| 42 | Ferulic acid+SO_3_ | C_10_H_10_O_7_S | 6.03 | 273.0071 | 273.0069 | 0.73 | 4888 | 193.0511 [M-H-SO_3_]^-^  149.0604 [M-H-SO_3_-CO_2_]^-^  134.0372 [M-H-SO_3_-CH_3_]^-^ | M |
| 43 | Ferulic acid-CH_2_O+2H+SO_3_ | C_9_H_10_O_6_S | 6.17 | 245.0137 | 245.0120 | -6.94 | 1402 | 165.0564 [M-H-SO_3_]^-^  121.0680 [M-H-SO_3_-CO_2_]^-^ | M |
| 44 | Caffeic acid+4H | C_9_H_12_O_4_ | 6.97 | 183.0660 | 183.0657 | 1.64 | 8222 | 165.0557 [M-H-H_2_O]^-^  147.0455 [M-H-2H_2_O]^-^ | M |
| 45 | Caffeic acid-O+SO_3_ | C_9_H_8_O_6_S | 6.99 | 242.9973 | 242.9963 | 4.12 | 1140 | 163.0400 [M-H-SO_3_]^-^  119.0520 [M-H-SO_3_-CO_2_]^-^ | M |
| 46 | Ferulic acid+4H | C_10_H_14_O_4_ | 7.19 | 197.0818 | 197.0814 | 2.03 | 1759 | 179.0703 [M-H-H_2_O]^-^ | M |
| **Coumarins** | | | | | | | | | |
| 47 | Esculetin+C_6_H_8_O_6_ | C_15_H_14_O_10_ | 4.18 | 353.0502 | 353.0509 | -1.98 | 1869 | 177.0193 [M-H-C_6_H_8_O_6_-H_2_O]^-^,  133.0273 [M-H-C_6_H_8_O_6_-CO_2_]^-^ | M |
| 48 | Esculetin+SO_3_ | C_9_H_6_O_7_S | 4.65 | 256.9759 | 256.9756 | 1.17 | 1070 | 177.0193 [M-H-SO_3_]^-^,  133.0273 [M-H-SO_3_-CO_2_]^-^ | M |
| 49 | Esculetin | C_9_H_6_O_4_ | 6.78 | 177.0190 | 177.0188 | 1.13 | 1683 | 133.0273 [M-H-CO_2_]^-^ | P |
| 50 | Esculetin+SO_3_ | C_9_H_6_O_7_S | 6.54 | 256.9760 | 256.9756 | 1.56 | 1598 | 177.0193 [M-H-SO_3_]^-^,  133.0273 [M-H-SO_3_-CO_2_]^-^ | M |

Table S2 (Continued)

| **NO.** | **Identification** | **Formula** | ***t*_R_ (min)** | **[M+H]^+^/[M-H]^-^** | | **Error (ppm)** | **Response** | **MS/MS** | **Type** |  |  |  |
| --- | --- | --- | --- | --- | --- | --- | --- | --- | --- | --- | --- | --- |
|  |  |  |  | **Meas. (*m/z*)** | **Pred. (*m/z*)** |  |  |  |  |  |  |  |
| **Diterpene lactones** | | | | | | | | | |  |  |  |
| 51 | Andrographolide+2H+CH_2_+SO_3_ | C_21_H_34_O_8_S | 8.81 | 445.1881 | 445.1896 | -3.37 | 705 | 365.2324 [M-H-SO_3_]^-^ | M |  |  |  |
| 52 | 12-Hydroxyandrographolide+CH_2_ | C_21_H_34_O_6_  5-Hydroxysparteine | 16.91 | 381.228 | 381.2277 | 0.79 | 2604 | 363.2146 [M-H-SO_3_-H_2_O]^-^  301.2195 [M-H-SO_3_-2H_2_O-CO_2_]^-^ | M |  |  |  |
| 53 | 14-Deoxyandrographolide+CH_2_+SO_3_ | C_21_H_32_O_7_S | 19.2 | 427.1789 | 427.1790 | -0.23 | 2393 | 347.2224 [M-H-SO_3_]^-^  303.2323 [M-H-SO_3_-CO_2_]^-^ | M |  |  |  |
| 54 | 14-Deoxyandrographolide+CH_2_ | C_21_H_32_O_4_ | 21.24 | 347.2221 | 347.2222 | -0.29 | 4530 | 303.2323 [M-H-SO_3_-CO_2_]^-^ | M |  |  |  |
| 55 | 14-Deoxyandrographolide+CH_2_+SO_3_ | C_21_H_32_O_7_S | 21.22 | 427.1789 | 427.1790 | -0.23 | 2393 | 347.2224 [M-H-SO_3_]^-^  303.2323 [M-H-SO_3_-CO_2_]^-^ | M |  |  |  |

Table S2 (Continued)

| **NO.** | **Identification** | **Formula** | ***t*_R_ (min)** | **[M+H]^+^/[M-H]^-^** | | **Error (ppm)** | **Response** | **MS/MS** | **Type** |  |  |  |
| --- | --- | --- | --- | --- | --- | --- | --- | --- | --- | --- | --- | --- |
|  |  |  |  | **Meas. (*m/z*)** | **Pred. (*m/z*)** |  |  |  |  |  |  |  |
| Anthraquinones | | | | | | | | | |  |  |  |
| 56 | Aloin-C_6_H_10_O_5_-2H+O+C_6_H_8_O_6_ | C_21_H_18_O_11_ | 11.19 | 445.0771 | 445.0771 | 0.00 | 1065 | 269.0450 [M-H-C_6_H_8_O_6_]^-^  240.0406 [M-H-C_6_H_8_O_6_-CHO]^-^  211.0390 [M-H-C_6_H_8_O_6_-2CHO]^-^ | M |  |  |  |
| 57 | Aloin-C_6_H_10_O_5_+O+C_6_H_8_O_6_ | C_21_H_20_O_11_ | 12.91 | 447.0923 | 447.0927 | -0.89 | 1953 | 271.0598 [M-H-C_6_H_8_O_6_]^-^  240.0406 [M-H-C_6_H_8_O_6_-CH_2_OH]^-^  255.0669 [M-H-C_6_H_8_O_6_-O]^-^ | M |  |  |  |
| 58 | 10-Hydroxyaloin | C_21_H_22_O_10_ | 17.15 | 433.1135 | 433.1135 | 0.00 | 2204 | 270.0536 [M-H-C_6_H_11_O_5_]^-^  253.0472 [M-H-C_6_H_11_O_5-_OH]^-^  253.0404 [M-H-C_6_H_11_O_5_-H_2_O]^-^ | P |  |  |  |
| Chromones | | | | | | | | | |  |  |  |
| 59 | Aloeresin D-C_15_H_16_O_7_+4H | C_14_H_20_O_4_ | 11.24 | 251.1281 | 251.1283 | -0.80 | 1579 | 149.0603 [M-H-C_5_H_10_O_2_]^-^ | M |  |  |  |
| 60 | Aloeresin D-C_15_H_16_O_7_+4H | C_14_H_20_O_4_ | 19.11 | 251.1281 | 251.1283 | -0.80 | 2047 | 149.0603 [M-H-C_5_H_10_O_2_]^-^  121.0632 [M-H-C_6_H_10_O_3_]^-^ | M |  |  |  |

**P: prototype components, M: metabolites.**

## Animal Model Establishment

The rat model of CPID was established by implanting extraneous materials. Female SD rats were anesthetized by intraperitoneal injection of 5 g·L^-1^ sodium pentobarbital at a dose of 30 mg/kg, then fixed on the operating table, and the skin was disinfected in the middle of the abdomen. A long 2 cm incision was made in the middle of the lower abdomen, cut the skin along the midline of the abdomen, entered the abdominal cavity to find and fully expose the uterus, and made a transverse incision along the 1 cm of the left uterine horn. A plastic tube (diameter 2 mm, long 0.5 cm, heavy 5 mg) after alcohol disinfection was placed in the uterus, sutured and fixed with the uterine incision, and the uterus was returned to its original position. The abdominal wall and skin were continuously sutured with 5-0 silk thread, and the abdomen was closed at the end of the operation. The animals were fed normally after operation.

## Reversal effect of KFXYS on inflammatory indexes in CPID rats

**Sample Collection and Preparation** After taken blood from the abdominal aorta of rats, taken the uterus immediately, weighed the weight and record; taken 100mg uterus tissue, added 10 times the amount of PBS solution, homogenate, centrifuged at 9500 *×g* for 10 min at 4℃, the supernatant was taken and then stored at -80℃. The rest of the uterus tissue was fixed in 10% neutral formalin solution for HE staining. The ELISA kit was used to measure the levels of IL-6, TNF-α, IL-1 and IL-10 in uterine tissue homogenate to reflect the degree of inflammation. The organ index was calculated by Eqs. (1).

Organ index= Organ weight/Rat weight ×100% (1)

**Statistical Analysis** SAS 9.4 statistical software package was used for statistical analysis. All data are presented as mean ± SD. Differences between multiple groups were examined using the one-way ANOVA. The LSD-t test was used to compare the homogeneity of variance between the two groups, and Kruskal-Wallis test was used to compare the heterogeneity of variance between the two groups. The significance levels were *p* < 0.05.

## Metabolomics analysis

**UPLC-Q-TOF/MS Analysis**

The capillary voltage in positive and negative ion mode were +3 KV and -2.2 KV, respectively; the desolvation gas temperature, 400℃; desolvation gas flow, 800 L·h^-1^.

The serum polarity part analysis condition: in positive ion mode, the mobile phase composed of acetonitrile-water (95:5, v/v) containing 10 mM ammonium formate (A) and water containing 10 mM ammonium formate (B), PH=3. In negative ion mode, the mobile phase composed of acetonitrile-water (95:5, v/v) containing 10 mM ammonium acetate and 0.1% ammonia (A) and water containing 0.1% ammonia (B), PH = 9. The elution program was as follows: 0-1.0min, 100% A; 1.0-3.0 min, 100%-90% A; 3.0-6.0 min, 90%-70% A; 6.0-8.0 min, 70%-50% A; 8.0-9.0 min, 50% A; 9.0-9.1 min, 50%-100% A; 9.1-11 min, 100% A. The flow rate was set to 0.30 mL·min^-1^, and the injection volume were 2 μL and 3 μL in positive and negative ion mode, respectively. The Mass condition were as follows: cone gas flow, 20 L·h^-1^; ion source temperature, 100 ℃; the collision energy, 15-45 V. Data were collected between 50 and 1200 *m/z*.

The serum polarity part analysis condition: the mobile phase consisted of water-acetonitrile (2:3, v/v) containing 10 mM ammonium acetate (A) and acetonitrile-isopropanol (1:9, v/v) containing 10 mM ammonium acetate (B). The elution program was as follows: 0-2.0 min, 60 %-57 % A; 2.0-2.1 min, 57 %-50 % A; 2.1-12 min, 50 %-46 % A; 12-12.1 min, 46 %-30 % A; 12.1-18 min, 30 %-1 % A; 18-18.1 min, 1 %-60 % A; 18.1-20 min, 60 % A. The flow rate was set to 0.30 mL·min^-1^, and the injection volume were 2 μL and 4 μL in positive and negative ion mode, respectively. The Mass condition were as follows: cone gas flow, 30 L·h^-1^; ion source temperature, 120℃; the collision energies of positive and negative ion modes were 15-45 V and 15-50 V, respectively.
